# New Functional Alkoxysilanes and Silatranes: Synthesis, Structure, Properties, and Possible Applications

**DOI:** 10.3390/ijms241813818

**Published:** 2023-09-07

**Authors:** Sergey N. Adamovich, Arailym M. Nalibayeva, Yerlan N. Abdikalykov, Igor A. Ushakov, Elizaveta N. Oborina, Igor B. Rozentsveig

**Affiliations:** 1 A.E. Favorsky Irkutsk Institute of Chemistry, Siberian Branch of the Russian Academy of Sciences, 1 Favorsky Street, 664033 Irkutsk, Russia; 2 D.V. Sokolsky Institute of Fuel, Catalysis and Electrochemistry, 142 Kunayev Street, 050010 Almaty, Kazakhstan

**Keywords:** 3-aminopropyltriethoxysilane, 3-aminopropylsilatrane, acrylates, aza-Michael reaction, complexation, sorption

## Abstract

The aza-Michael reaction of 3-aminopropyltriethoxysilane (**1**) and -silatrane (**2**) with acrylates affords functionalized silyl-(**3**–**8**) and silatranyl-(**9**–**14**) mono- and diadducts with up to a 99% yield. Their structure has been proved with IR and NMR spectroscopies, mass spectrometry and XRD analysis. The hydrolytic homo-condensation of triethoxysilanes **3**–**5** gives siloxanes **3a**–**5a**, which form complexes with Ag, Cu, and Ni salts. They are also able to adsorb these metals from solutions. The hetero-condensation reaction of silanes **4**–**8** with OH groups of zeolite (**Z**), silica gel (**S**) and glass (**G**) delivers the modified materials (**Z4**, **S7**, **G4**, **G5**, **G7**, **G8**, etc.), which can adsorb ions of noble metal (Au, Rh, Pd: **G4** + **Au**, **G5** + **Pd**, **G7** + **Rh**). Thus, the synthesized Si-organic polymers and materials turned out to be promising sorbents (enterosorbents) of noble, heavy, toxic metal ions and can be applied in industry, environment, and medicine.

## 1. Introduction

**Scheme 1:**
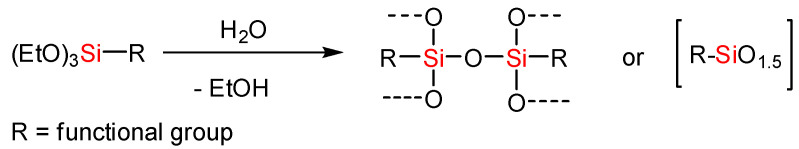
Homo-condensation reaction of functional triethoxysilanes.

Functional 1-organyltrialkoxysilanes, R-Si(OAlk)_3_, are well-known organosilicon compounds. They have found applications in materials science and various technologies as building blocks, hybrid- and nano-materials, optical sensors, solar cells, catalysts, silicone sealants, and adhesives. These materials are formed via the homo-condensation reaction to afford siloxanes or silsesquioxanes, [R-SiO_1.5_], (Scheme 1) [1,2,3,4].

Such carbofunctional siloxanes are efficient ingredients of rubber mixtures for non-combustible, water- and wear-resistant tires, as well as for ion-exchange and complex-forming sorbents of heavy and noble metals.

Carbofunctional polyorganylsilsesquioxanes, like siloxanes, are also excellent sorbents. They are easily synthesized and their composition and properties can be readily reproduced. The spatially cross-linked silsesquioxane structure of these sorbents ensures their high thermal stability and resistance to highly aggressive acidic media. At the same time, the silsesquioxane matrix does not affect the specific reactivity of their carbofunctional substituents. An important application of carbofunctional polyorganylsilsequioxanes and initial organosilicon monomers is in the design of a new generation of “test systems”. In economic terms, it is more rational to use inexpensive natural zeolites and silicas. However, such materials are not as efficient in the extraction of the target components, for example, noble metals. More expensive synthetic sorbents, primarily carbofunctional polyorganylsilsesquioxanes, due to a wide range of reactive groups that can be easily introduced into their composition, are characterized by higher efficiency and wider application scope [5,6].

**Scheme 2:**
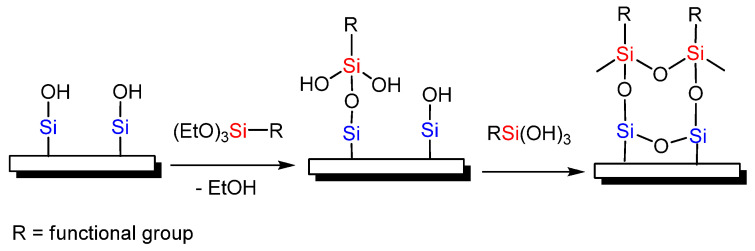
Modification of silica gel with functional triethoxysilanes (hetero-condensation reaction).

In addition, functional 1-organyltrialkoxysilanes are employed in the modification (silanization) of hydroxylated surfaces (silicon oxide, alumina, quartz, mica, zeolite, glass, etc.) via hetero-condensation (grafting) to give self-assembled monolayers (SAMs) (Scheme 2). 

The interest in SAM is due to their potential application as functional modules with tailor-made physical and chemical properties. This is especially true for the use of SAM in surface engineering. For example, the advantages of unique properties and complex architecture of SAM are especially pronounced in analytical chemistry. An effective technology is one for the formation of a pattern on the surface of semiconductors. In addition, engineered surfaces can improve adhesion. Such surfaces (modified materials) can be used as heterogeneous catalysts, special filters/membranes, and, like functional siloxanes, as effective sorbents/enterosorbents of heavy or toxic metals [7,8].

**Scheme 3:**
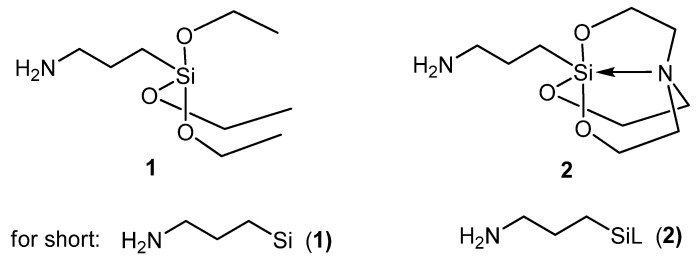
Chemical structures of 3-aminopropyltriethoxysilane (**1**) and 3-aminopropylsilatrane (**2**).

All of the above is true both for 3-aminopropyltriethoxysilane, NH_2_-(CH_2_)_3_-Si(OCH_2_CH_3_)_3_ (**1**), and 3-aminopropylsilatrane, NH_2_-(CH_2_)_3_-Si(OCH_2_CH_2_)_3_N (**2**) (Scheme 3) [9,10].

As seen from Scheme 3, compounds **1** and **2** are close analogues. However, it should be emphasized that silatranes (unlike silanes and siloxanes) possess high and diverse biological and pharmacological activity [11]. For example, silatrane **2** and its derivatives exhibit a growth-stimulating effect on agricultural crops (wheat, rye, oats, corn, etc.) [12,13]. They are efficient antifungal (*Aspergillus fumigatus*, *Penicillium chrysogenum*, *Fusarium*) [14], antimicrobial (*Enterococcus durans*, *Bacillus subtilis*, *Escherichia coli*, *Pseudomonas aeruginosa*, *Hepatitis B virus*) [15,16,17], antiparasitic (*Giardia lamblia*, *Trichomonas vaginalis*) [18] and anticancer (adenocarcinoma, hepatocarcinoma, melanoma) agents [19,20] and potential drugs [21].

Among nucleophilic addition processes, the aza-Michael reaction holds a special place [22,23,24]. This general reaction proceeds with the participation of various Michael donors (carbamates, amides, amines) and Michael acceptors (unsaturated nitriles, sulfones, phosphonates, and also acrylates). Acrylates and acrylate polymers are known to be functional compounds and materials. The latter have valuable characteristics such as low cost, transparency, thermoplasticity, stability, biocompatibility, and high adhesion to metals [25]. In particular, methacrylate-functionalized silanes and silatranes are used to produce hybrid materials and in sol-gel processes and coating technologies [26,27].

The amino group, which is contained in compounds **1** and **2**, makes them functional and allows them to be involved into the aza-Michael reaction. It was reported that some aminoalkoxysilanes can be added via the aza-Michael process [28,29]. However, to the best of our knowledge, the participation of 3-aminopropylsilatrane **2** in this reaction was described only in one work [30] on the example of addition to N-phenylmaleimide.

In order to expand the scope of polyfunctional Si-organic compounds and materials, here we have implemented the aza-Michael reaction of silane **1** and silatrane **2** with various acrylates and have studied the adsorption activity of the target adducts and their derivatives.

## 2. Results and Discussions

### 2.1. Synthesis

**Scheme 4:**
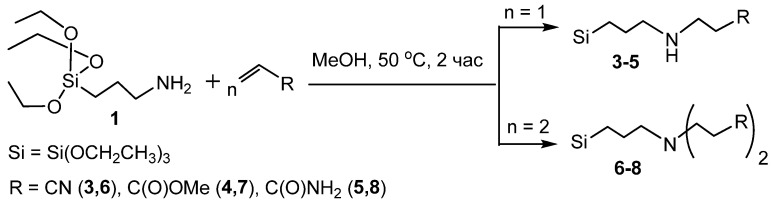
Synthesis of monoadducts **3**–**5** and diadducts **6**–**8**; Si = Si(OCH_2_CH_3_)_3_.

**Scheme 5:**
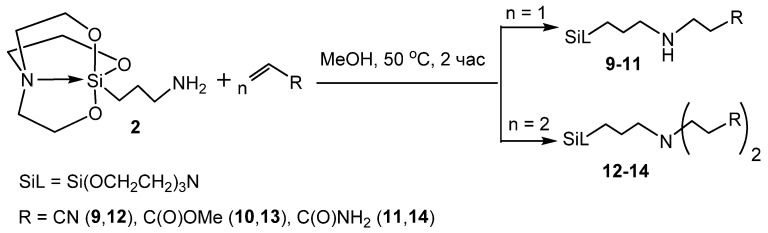
Synthesis of monoadducts **9**–**11** and diadducts **12**–**14**; SiL = Si(OCH_2_CH_2_)_3_N.

The synthesis of silane **1** and silatrane **2** derivatives is shown in Scheme 4 and Scheme 5. The target monoadducts **3**–**5** and **9**–**11** and diadducts **6**–**8** and **12**–**14** were obtained using the aza-Michael reaction with electron-deficient alkenes (acrylonitrile, methyl acrylate and acrylamide).

Using the reaction of silane **1** and silatrane **2** with acrylonitrile and varying solvents (CHCl_3_, MeCN, THF, MeOH, EtOH), temperature (20–80 °C) and time (2–48 h), we found the following optimum conditions for the synthesis of silanes **3** and **6** (yields 99% and 16%): methanol, 50 °C, 2 h and 48 h, respectively. The best conditions for the preparation of silatranes **9** and **12** (yields 99% and 97%) were as follows: methanol, 50 °C, 1 h and 4 h, respectively.

The higher reactivity of silatrane **2** compared to silane **1** may be due to the significant dipole moment (μ = 5.3–7.1 D) and polarity as well as the strong electron-donating effect of the silatranyl and silatranylmethyl groups (inductive constants σI = −0.56, σ∗ = −3.49 and σI = −0.36, σ∗ = −2.24). At the same time, the triethoxysilyl group had electron-acceptor properties [31,32,33].

### 2.2. Spectroscopic and Spectrometric Studies

The composition and structure of the obtained compounds were confirmed with IR, NMR spectroscopy, mass spectrometry and elemental and X-ray diffraction analysis (see Section 3 and Supplementary Materials).

#### 2.2.1. IR Spectroscopy

The IR spectra of compounds **3**–**14** show characteristic absorption bands at 763–770 cm^−1^, 1090–1103 cm^−1^ (Si-O) and 2925–2939 cm^−1^ (ν_s_ CH_2_). The spectra of monoadducts contain absorption bands (ν_s_ C-NH) and (ν_s_ NH) at 1392–1421 cm^−1^ and 3310–3447 cm^−1^, respectively. In the spectra of compounds **3**, **6**, **9** and **12**, the absorption bands were observed at 2190–2236 cm^−1^ (C≡N). The spectra of carbonyl-containing mono- and diadducts displayed characteristic absorption bands in the region 1671–1736 cm^−1^ (C=O). In addition, absorption bands at 584–588 cm^−1^ (N → Si) were detected in the spectra of all silatranes **9**–**11** and **12**–**14** (Figures S1–S4).

#### 2.2.2. NMR Spectroscopy

NMR spectra (^1^H, ^13^C, ^15^N and ^29^Si) confirm the structure of both monoadducts **3**–**5** and **9**–**11** and diadducts **6**–**8** and **12**–**14** (Figures S5–S16). The NMR spectra of all compounds were recorded at room temperature. 

The ^1^H NMR spectra showed the characteristic triplets and quartets of the triethoxysilyl groups (CH_3_ and OCH_2_) in the regions of 0.5–06 ppm and 1.15–l.20 ppm (*J* = 7.0 Hz), respectively. The ^1^H NMR spectra contained the characteristic triplets of the silatranyl moiety representing the NCH_2_ and OCH_2_ groups at 2.73–2.79 ppm and 3.69–3.76 ppm (*J* = 5.9 Hz), respectively. The spectra of all compounds exhibited multiplets of the SiCH_2_, CH_2_, and NCH_2_ groups of propyl moiety at 0.30–0.41 ppm, 1.46–1.61 ppm and 2.41–2.52 ppm, respectively. In the spectra of all compounds, multiplets of the CH_2_, and CH_2_N groups of ethyl moiety were observed at 2.47–2.52 ppm and 2.84–2.90 ppm, respectively.

In the ^13^C NMR spectra of the compounds synthesized, peaks of the triethoxysilyl (CH_3_ and OCH_2_) and silatranyl moiety carbons (NCH_2_ and OCH_2_) were detected at 17.0–18.0 ppm and 56.50–58.80 and 50.59–51.21 ppm and 56.30–57.86 ppm, respectively. The spectra of all compounds showed peaks of the propyl moiety carbons (SiCH_2_, CH_2_ and NCH_2_) at 12.80–13.80 ppm, 16.20–24.22 ppm and 25.15–33.90 ppm, respectively, as well as peaks of the ethyl moiety carbons (CH_2_ and CH_2_N) at 52.60–53.00 ppm and 54.00–54.20 ppm, respectively.

The ^15^N NMR spectra demonstrate signals of the nitrogen at ∼−135 ppm (C≡N), −250 ppm (NH), −340 ppm (N) and −360 ppm (NSilatrane).

The ^29^Si NMR spectra of all silatranes contained peaks of the silatranyl moiety at ∼−85 ppm. Such δ values indicate an intramolecular transannular Si ← N dative bond [15,18]. 

#### 2.2.3. Mass Spectrometry

We failed to record high resolution mass spectra of silanes **3**–**8**, possibly due to their hydrolytic instability. However, the spectra of silatranes confirmed their composition and structure. For example, the mass spectrum of compound **9** contained the main peak in the form of the [M + H]^+^ ion. The theoretical *m*/*z* value was 286.158695; in reality, a value of 286.15876 was obtained, so the error was 0.2 ppm. The mass spectrum of silatrane **12** was registered in the form of the ion [M + H]^+^ with *m*/*z* 339.18498 (theoretical *m*/*z* value was 339.185244); the error was 0.8 ppm.

#### 2.2.4. X-ray Spectrometry

**Figure 1:**
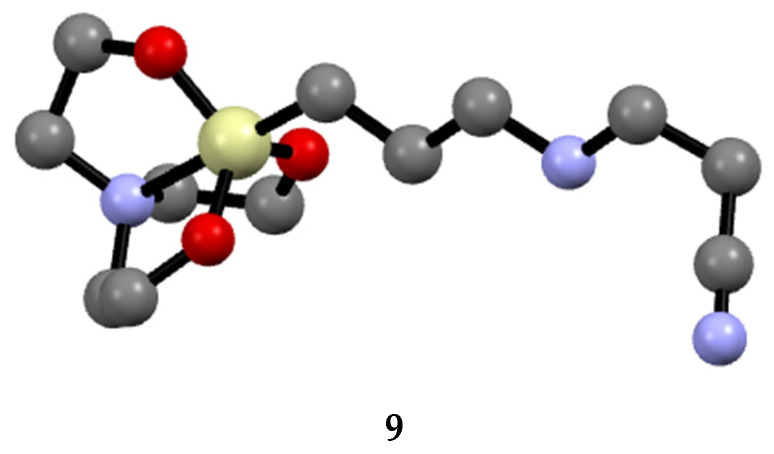
Molecular structure of compound **9**.

The structure of compound **9** was proved with X-ray diffraction analysis. Single crystals of **9** were grown from CHCl_3_ solution. The molecular structure is depicted in Figure 1.

Atomic coordinates, bond lengths, bond angles and atomic displacement parameters for the crystal structure of **9** have been deposited with the Cambridge Crystallographic Data Centre (CCDC) with the deposition number CCDC 2263862. These data can be obtained free of charge from the CCDC via https://www.ccdc.cam.ac.uk/structures/ (accessed on 18 May 2023).

### 2.3. Functional Materials

#### 2.3.1. Polyorganosiloxanes Sorption

**Scheme 6:**
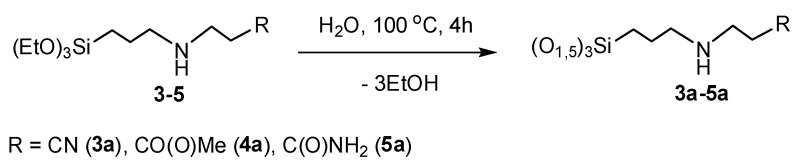
Synthesis of polysiloxanes **3a**–**5a**.

The solid insoluble polyorganosiloxanes, R(CH_2_)_2_NH-(CH_2_)_3_-Si(O_1.5_)_3_ **3a**–**5a**, were synthesized using the hydrolytic homo-condensation (compare Scheme 1) of mono-substituted silanes R-(CH_2_)_2_NH-(CH_2_)_3_-Si(OCH_2_CH_3_)_3_ **3**–**5** (Scheme 6).

**Figure 2:**
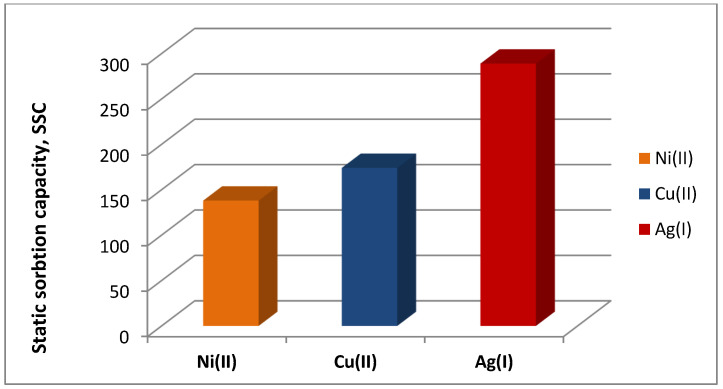
Calculated values of static sorption capacities (SCE) of polysiloxane **3a**.

Compounds **3**–**5** and **3a**–**5a** were preliminary studied as chelating ligands and potential sorbents of noble (AgI) and transition Cu(II) and Ni(II) metals. Monomers **3**–**5** turned out to be inefficient owing to their low stability. At the same time, extremely stable polymers **3a**–**5a** showed high activity (static sorption capacity, SSC = 138–289 mg/g), see, for example, Figure 2 and the Supplementary Materials (compare with SSC 29.5 mg/g, 24.9 mg/g and 16.6 mg/g for Cu(II), Co(II) and Ni(II), respectively [8]).

**Figure 3:**
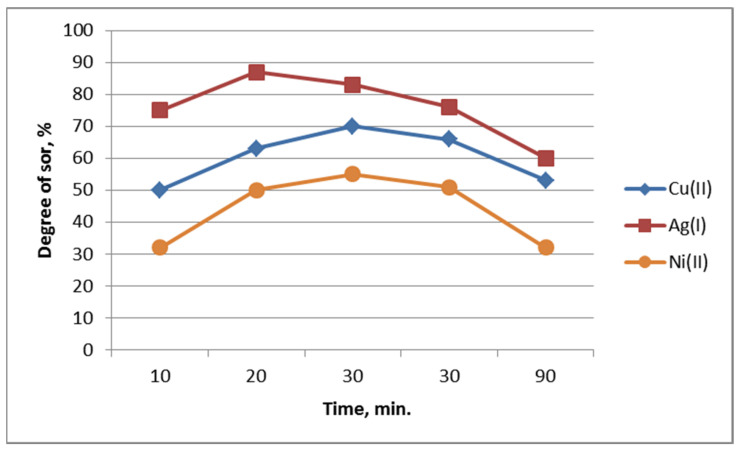
The effect of time on the degree of sorption of metals by polysiloxane **3a**.

The sorption equilibrium with respect to silver was reached for 20 min. For nickel and copper, this takes 30 min. Kinetic dependences were represented by typical curves, which had a steep section in the region of 10–30 min of the sorbate solution contact with the polymer; see, for example, Figure 3.

**Scheme 7:**
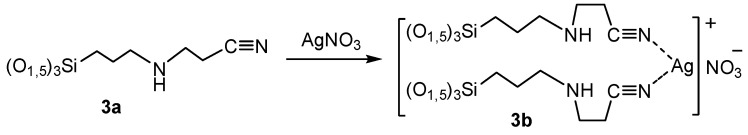
Formation of complex **3b**.

Mechanistically, the sorption likely occurs via the formation of carbonyl and nitrile complexes with Ag, Cu and Ni salts (for example, complex **3b**, Scheme 7).

The C≡N-Ag coordination was confirmed by IR spectroscopy. For instance, the IR spectrum of the initial powder **3a** showed the absorption peak at 2236 cm^−1^, which corresponded to C≡N stretching vibrations. In the case of **3b** with the additive of AgNO_3_, the absorption peak shifted and appeared at 2257 cm^−1^ (∆ν = 21 cm^−1^). This indicates that the C≡N group of polymer **3b** interacted with the Ag^+^ ion. The IR spectrum of the starting methylacrylate silsesquioxane, MeO(O)C(CH_2_)_2_NH-(CH_2_)_3_-Si(O_1.5_)_3_ **4a**, contained a band at 1700 cm^−1^, which was assigned to the stretching vibration of the C=O bond. IR spectra **4b** showed clear shifts in the C=O stretching vibration to 1690 cm^−1^ and 1681 cm^−1^, ∆ν = 10 cm^−1^ and 19 cm^−1^, respectively, after the addition of NiCl_2_ and CuCl_2_.

A similar picture could be observed for the metal complexes of acrylamide silsesquioxane H_2_N(O)C(CH_2_)_2_NH-(CH_2_)_3_-Si(O_1.5_)_3_ **5a**, in which the metal was also coordinated through the carbonyl oxygen: for **5a** at 1670 cm^−1^ (C=O); for **5a** + NiCl_2_ at 1660 cm^−1^ (C=O); and for **5a** + CuCl_2_ at 1650 cm^−1^ (C=O).

The analysis of our own data and those from the literature demonstrates that the interaction of metal ions (Ag, Cu, Ni) with C≡N and C=O functional groups of polyorganosilsesquioxanes **3a**–**5a** is thermodynamically favorable [34]. It occurs via chelate interaction and is accompanied by the formation of 2:1 complexes (for example, Scheme 7) [35,36].

Thus, polymers **3a**–**5a** are prospective chemically and thermally stable sorbents (enterosorbents) of heavy and toxic metal ions and can be used in industry, the environment and medicine.

#### 2.3.2. Modification of Hydroxylated Surfaces’ (Zeolite, Silicagel, Glass) Sorption

Functionalized materials find wide applications in the fields of catalysis, separation, sensors, optoelectronics, environmental technology, and biomedical applications due to their high specific surface area and chemical, thermal and mechanical stability [7,8].

The high density of OH groups, for example, on the silica surfaces, promotes the covalent grafting of useful functional groups onto the interior and exterior surfaces via hetero-condensation reactions (see Scheme 2). Functional 1-organyltrialkoxysilanes are very often used to modify hydroxylated surfaces. The organic–inorganic skeletons thus obtained can exhibit significant hydrophobicity, high adsorption activity, and selectivity compared to the starting materials [9,10].

Using the synthesized 1-organyltrialkoxysilanes **4**–**8** containing C≡N, C=O, NH and H_2_N groups, a series of preliminary experiments on the modification (silanization) of zeolite (**Z**), silica gel (**S**) and glass (**G**) surfaces was carried out. The degree of modification of zeolite “heulandite” **Z** with silane **4** was estimated from the increase in the mass of modified zeolite (**Z4**). The content of the modifier in **Z** increased linearly at higher concentrations of silane **4** solution (Table 1).

**Figure 4:**
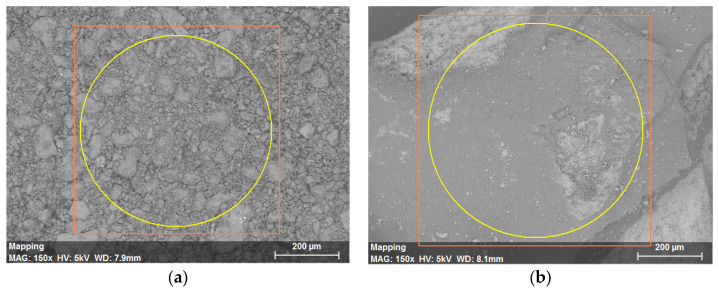
SEM images: (**a**) **Z**; (**b**) **Z4**. Red box—scan area, yellow circle—reading area.

The SEM-EDX analysis was performed to confirm the fixation of modifier **4** on the surface of the zeolite **Z**. SEM images of **Z** and **Z4** are shown in Figure 4.

The surface morphology of natural zeolites, including heulandite **Z**, is rather heterogeneous due to the coexistence of various crystalline phases (Figure 4a). The chemical modification of **Z** changes the surface structure of the material (Figure 4b). After modification, the surface of **Z** becomes more uniform and less porous. This indicates a high degree of the zeolite coating with the modifier layer.

**Figure 5:**
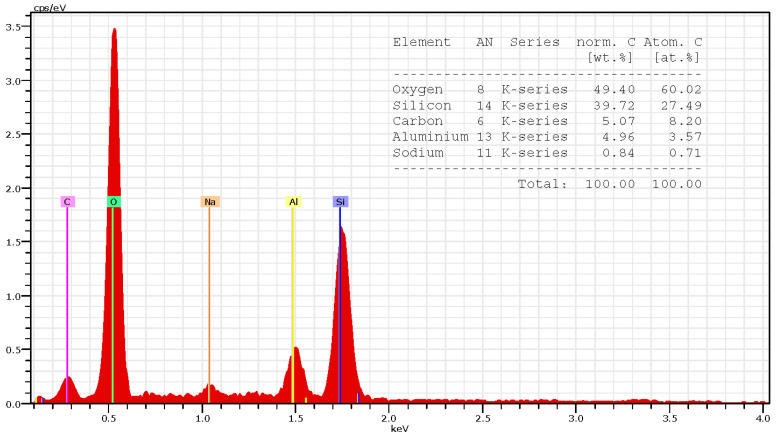
EDX spectrum and elemental composition of the initial **Z**.

**Figure 6:**
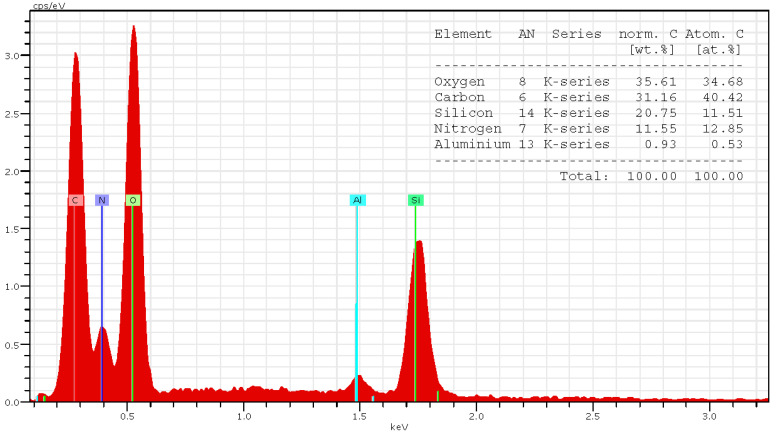
EDX spectrum and elemental composition of modified zeolite **Z4**.

The high degree of **Z** coating with modifier **4** was also confirmed by the EDX analysis (Figure 5 and Figure 6). The increased carbon (31 wt.% vs. 5 wt.%) and nitrogen (11 wt.% vs. 0 wt.%) content, and the practical absence of aluminum and sodium on the **Z4** surface, indicate that the zeolite surface in the studied area was covered with a layer of **4**.

A similar picture was observed when silica gel (**S**) and glass (**G**) were treated with silanes **7** and **4**. Modified surfaces **S7** and **G4** were formed, respectively. 

EDX scanning and elemental composition showed that the main elements of **S** were O 64.05% and Si 33.95%; the main elements of **G** were O 63.57%, Si 31.45% and C 4.98%, respectively. The surface of the modified **S7** and **G4** consisted of O 48.35%, Si 29.01% and C 22.64%, and O 44.35%, C 32.32%, Si 14.86% and N 8.47%, respectively. The high carbon (22–34% vs. 0–5%), and nitrogen (4.5–13% vs. 0%) content on the **S7**, **G4**, **G5** and **G7**, **G8** surface shows that the silica gel and glass surfaces were covered with silane layers (Figures S20–S25).

The hetero-condensation reaction (Scheme 2) allowed the surfaces of zeolite, silica gel and glass modified with functional silanes (C≡N, C=O, NH and NH_2_ groups) to be obtained. According to Pearson’s hard and soft acid–base theory, compounds (polymers) containing functional groups with N or O donor atoms should be prospective sorbents of precious metal ions. Precious metals are widely used in many fields, e.g., in the electrical and electronics industry, various chemical processes, the manufacture of catalysts and the production of corrosion resistant materials and jewelry.

Therefore, model samples of glass modified with functional silanes were used for a preliminary evaluation of the noble metals’ sorption from aqueous solutions of AuCl_3_, RhCl_3_ and PdCl_2_. The sorption solutions have equal concentrations of each metal 5 mg/25 mL (square of glass 1 cm^2^, contact time 4 h, pH = 2–5, temperature 25 °C). After sorption from solutions, samples of modified glasses (**G4**, **G5**, **G7** and **G8**) were isolated, washed (ethanol, water) and dried (100 °C, 4 h). The EDX analysis of these samples enabled an estimation of the degree of metal ions retained by these glasses from solutions.

EDX spectra and the mapping of metals on the surfaces of **G4**, **G5**, **G7** and **G8** (+**Au**, +**Pd**, +**Rh**) are shown in Figure 7 and Figure 8 and Figures S26–S34.

**Figure 7:**
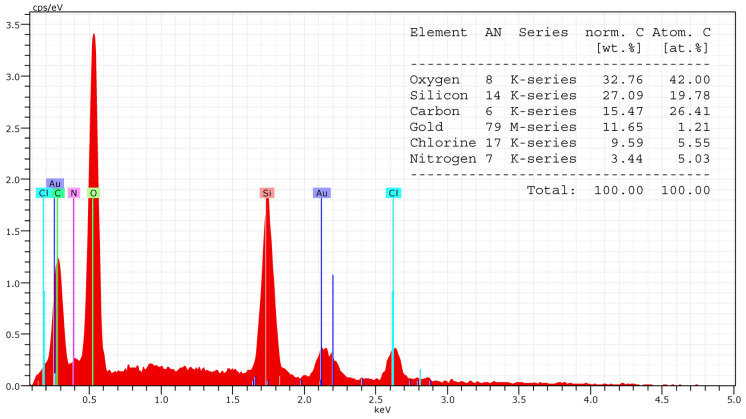
EDX spectrum and elemental composition of (**G4** + **Au**).

The samples contained Au 11–15 wt.%, Pd 6–14 wt.% and Rh 0.8–6 wt.% (Figure 7 and Figures S26–S34). The content can be compared with Cu 2.28 wt.%, Co 2.13 wt.% and Ni 0.95 wt.% [8]. This indicates that the modified glasses are good sorbents for the extraction of these metals. Moreover, glasses coated with monosilanes (**G4** and **G5**) showed the best results. Metal adsorption increased in the series Rh < Pd < Au and did not change at pH = 2, 3 and 5. 

The adsorption of Au(III), Rh(III) and Pd(II) may be due to the ionic mechanism of the interaction between protonated nitrogen atoms in **G4**, **G5**, **G7** and **G8** and chloranionic metal complexes, or due to the formation of chelate compounds of metal ions with donor N or O atoms. Both mechanisms are assumed to be probable [37].

Thus, we have demonstrated that the new sorbents have a high potential for the analysis and concentration of Au(III), Rh(III) and Pd(II).

## 3. Materials and Methods

### 3.1. Materials

3-Aminopropyltriethoxysilane (**1**), 3-aminopropylsilatrane (**2**), acrylonitrile, methylacrylate and acrylamide were purchased from Sigma-Aldrich (Merck KGaA, Darmstadt, Germany). The objects of study were natural zeolite of the Kholinsky deposit (Russia), calcium heulandite, Ca[Al_2_Si_7_O_18_]⋅6H_2_O (**Z**), commercial chromatography grade silica gel 60–120 Mesh (**S**) and slides glass (**G**) 1 cm × 1 cm.

The procedure earlier described was used to immobilize silanes on the zeolite (**Z**) surface [38]. Syntheses of silane-functionalized silica gel (**S**) and glass (**G**) were described in [39,40], respectively. Reagents of high or analytical purity were used for all sorption experiments. The 1 mg mL^−1^ Au(III), Rh(III) and Pd(II) standard stock solutions were prepared by dissolving pure HAuCl_4_·4H_2_O, RhCl_3_·3H_2_O and PdCl_2_ in dilute HCl, respectively [37].

### 3.2. Methods

The IR spectra were registered on a Varian 3100 FTIR spectrometer (Digilab LLC, Marlborough, MA, USA) in 4000–400 cm^−1^ range with the sample as a thin film or tablet (KBr).

The ^1^H, ^13^C and ^15^N NMR spectra were run in CDCl_3_ at room temperature on Bruker DPX-400 and AV-400 spectrometers (Bruker BioSpin GmbH, Rheinstetten, Germany) (400.13, 100.61 and 40.56 MHz, respectively). Chemical shifts were referred to TMS (^1^H, ^13^C) and nitromethane (^15^N). 

Mass spectra were recorded on an HR-TOF-ESI-MS Agilent 6210 with the registration mode of positive ions with acetonitrile as a solvent (in case of poor solubility by ultrasound) and 0.1% perfluorobutyric acid as an ionizing agent.

X-ray Diffraction Analysis. The single crystals of compounds **6** and **12** were grown by the slow evaporation of chloroform solutions at room temperature. The X-ray diffraction data were collected with a Bruker D8 VENTURE diffractometer (Bruker Optik GmbH, Ettlingen, Germany) (PHOTON III CMOS detector, Mo IµS3.0 X-ray source, Montel mirror-focused MoKα radiation λ = 0.71073 Å, N_2_-flow cryostat) via 0.5° ω- and φ-scan techniques. Data were corrected for absorption effects using the multi-scan method (SADABS) [41]. The structure was solved and refined using the Bruker SHELXTL Software Package [42].

Elemental analysis was performed on a Thermo Scientific Flash 2000 Elemental Analyzer (Thermo Fisher Scientific Inc., Milan, Italy). Melting points were determined on a Kofler Hot-Stage Microscope PolyTherm A apparatus (Wagner & Munz GmbH, München, Germany). 

The spectrophotometric determination of the elements was carried out on Specol-10 spectrophotometers and a KFK-2 photocolorimeter using appropriate techniques [43].

The morphology of the surfaces was determined with scanning electron microscopy using a Hitachi TM3000 electron microscope (Hitachi High-Technologies Corporation, Tokyo, Japan) with a magnification up to 30,000× and a resolution up to 25 nm. The experiments were performed at 5 kV. Surface elements were established by analysis of energy-dispersive X-ray spectra (EDX) (Quantax 70). The samples were scanned on a Quanta 200 FEI SEM-EXD electron microscope (Quanta 200 FEI, Hillsboro, OR, USA).

### 3.3. Chemistry

General Procedure for the Synthesis of **3**–**14**.

A mixture of silane **1** or silatrane **2** (1 mmol) and the corresponding acrylate (1 or 2 mmol) in 10 mL of methanol was stirred at 50 °C for 2–4 h under an inert atmosphere (N_2_) or in air. The solvent was removed under reduced pressure. The residue was washed many times with ether and dried to give products **3**–**14**.

#### 3.3.1. 3-[(3-(Triethoxysilyl)propyl)]-amino)propanenitrile (**3**)

Product **3** was isolated as a colorless oil with yield of 99%. ^1^H-NMR (CDCl_3_, 400 MHz), δ (ppm): 0.58 (m, 2H, SiCH_2_); 1.14 (t, *J* = 7.0 Hz, 9H, CH_3_); 1.52 (m, 2H, CH_2_); 2.43 (t, 2H, CH_2_N); 2.56 (t, 2H, NCH_2_); 2.84 (t, 2H, CH_2_); 3.73 (q, *J* = 7.0 Hz, 6H, OCH_2_). ^13^C-NMR (CDCl_3_, 100 MHz), δ (ppm): 7.50 (SiCH_2_); 17.93 (CH_3_); 18.37 (CH_2_CN); 22.92 (SiCH_2_CH_2_); 44.61 (CH_2_CH_2_CN); 51.41 (CH_2_); 58.00 (OCH_2_); 118.43 (CN). 

Silanes **4**–**8** have been described in [29].

#### 3.3.2. 3-[3-(2,8,9-Trioxa-5-aza-1-silabicyclo[3.3.3]undec-1-yl)-propyl]amino)propanenitrile (**9**)

Silatranes **9**–**14** have been described in [44].

## 4. Conclusions

In conclusion, it was found that the aza-Michael reaction of commercially available 3-aminopropyltriethoxysilane (**1**) and -silatrane (**2**) with various acrylates afforded the corresponding functionalized mono- and diadducts (**3**–**14**). 1-Organyltriethoxysilanes **3**–**5** and their polymers (siloxanes) **3a**–**5a** were studied as chelating ligands of noble and transition metals. It was established that stable functional polyorganosiloxanes **3a**–**5a** formed carbonyl and nitrile complexes with salts of Ag, Cu, Ni and could sorb these metals from solutions (static sorption capacity, SSC = 138–289 mg/g).

In addition, the synthesized silanes **4**–**8** were employed for the modification (silanization) of surfaces of zeolite (**Z**), silica gel (**S**), and glass (**G**) surfaces to produce modified materials: **Z4**, **S7**, **G4**, **G5**, **G7**, **G8**, etc. The latter were used to study the sorption of noble metals (AuCl_3_, RhCl_3_, PdCl_2_). EDX spectra and the mapping of metals proved the formation of layers **G4** + **Au, G5** + **Pd, G7** + **Rh** and others. The sorption of metals increased in the series Rh < Pd < Au. The samples contained Au (up to 15%), Pd (up to 14%) and Rh (up to 6%). Thus, synthesized Si-organic polymers and materials are promising chemically and thermally stable sorbents (enterosorbents) of noble, heavy, toxic metal ions and can be used in industry, the environment and medicine.

## Figures and Tables

**Figure 8:**
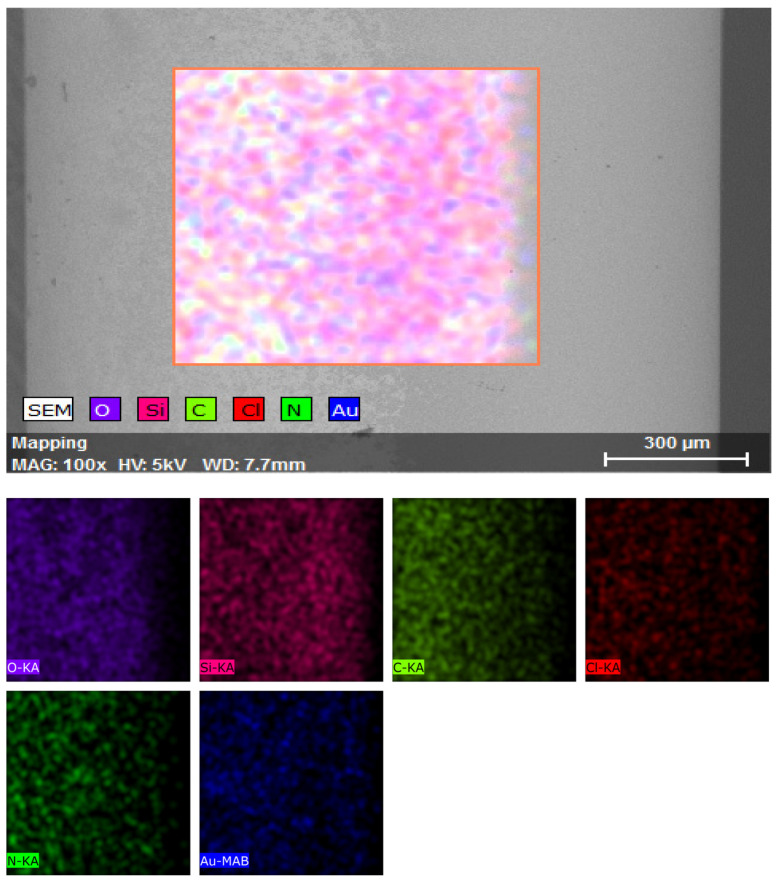
Elemental mapping of modified (**G4** + **Au**).

**Table 1:** Degree of **Z** modification with silane **4** (initial weight of **Z** 10 g, solvent: hexane).

Mass Silane 4 in Solution, %	Mass Z4, g	Mass Gain, g	Mass 4 in Z4, %
0.5	10.31	0.31	3.0
1.0	10.50	0.50	4.8
1.8	10.93	0.93	8.5
3.5	11.74	1.74	14.8
4.5	11.99	1.99	16.6
5.8	12.08	2.08	17.2

## Data Availability

The data presented in this study are available from the authors.

## Supplementary Materials

The following supporting information can be downloaded at: https://www.mdpi.com/article/10.3390/ijms241813818/s1.
