# Supplementary material for: New Functional Alkoxysilanes and Silatranes: Synthesis, Structure, Properties, and Possible Applications
Source: Int J Mol Sci. 2023 Sep 7;24(18):13818. doi: 10.3390/ijms241813818 (PMC10530882; doi:10.3390/ijms241813818)
Supplement: Supplementary file 1 [file ijms-24-13818-s001.zip › ijms-2580240-supplementary.pdf]

Supplementary Materials

New Functional Alkoxysilanes and Silatranes: Synthesis, Structure, Properties, and Possible Applications

Sergey N. Adamovich [1,*], Arailym M. Nalibayeva [2], Yerlan N. Abdikalykov [2], Igor A. Ushakov [1], Elizaveta N. Oborina [1], Igor B. Rozentsveig [1]

[1] A.E. Favorsky Irkutsk Institute of Chemistry, Siberian Branch of the Russian Academy of Sciences, 1 Favorsky Street, 664033 Irkutsk, Russia
[2] D.V. Sokolsky Institute of Fuel, Catalysis and Electrochemistry, 142 Kunayev Street, 050010 Almaty, Kazakhstan

IR spectra

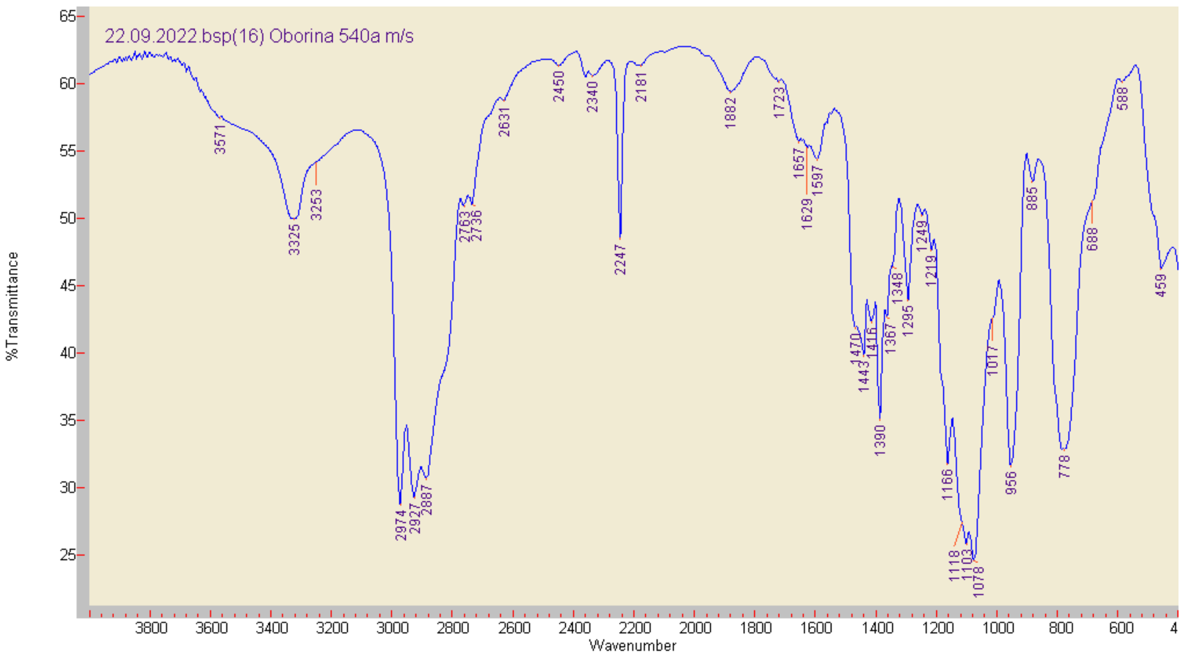

Fig. S1. IR spectrum of silane **3**

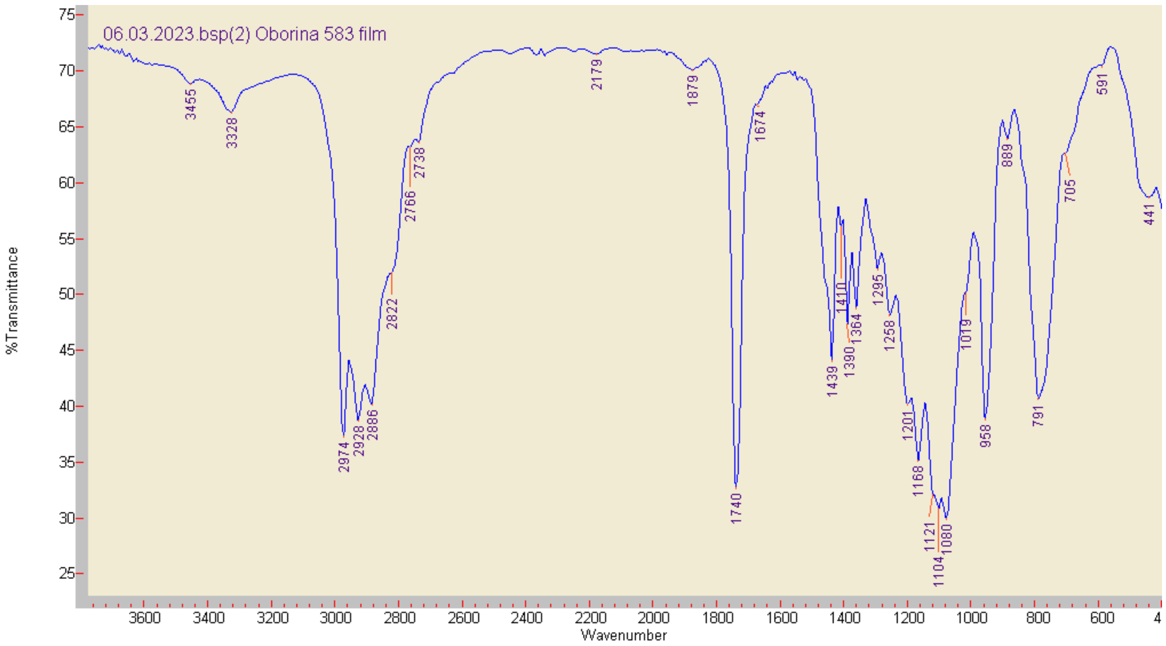

Fig. S2. IR spectrum of silane **4**

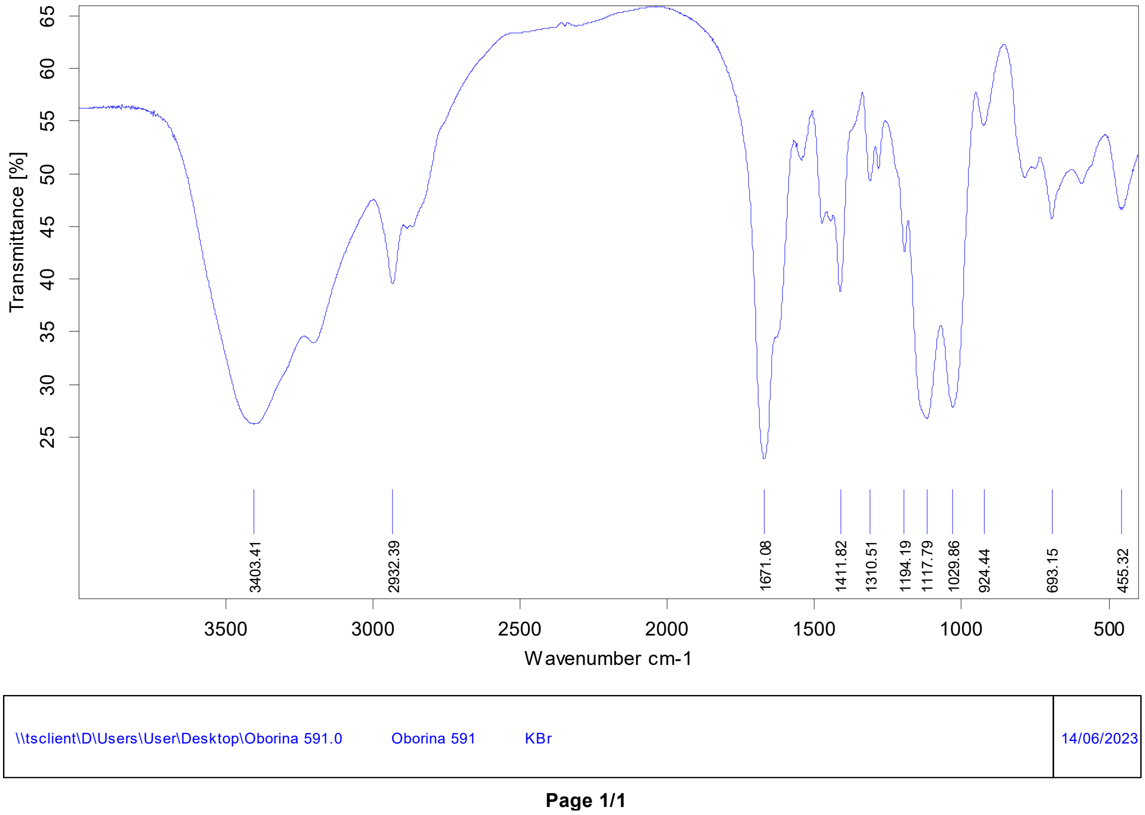

Fig. S3. IR spectrum of silane **5**

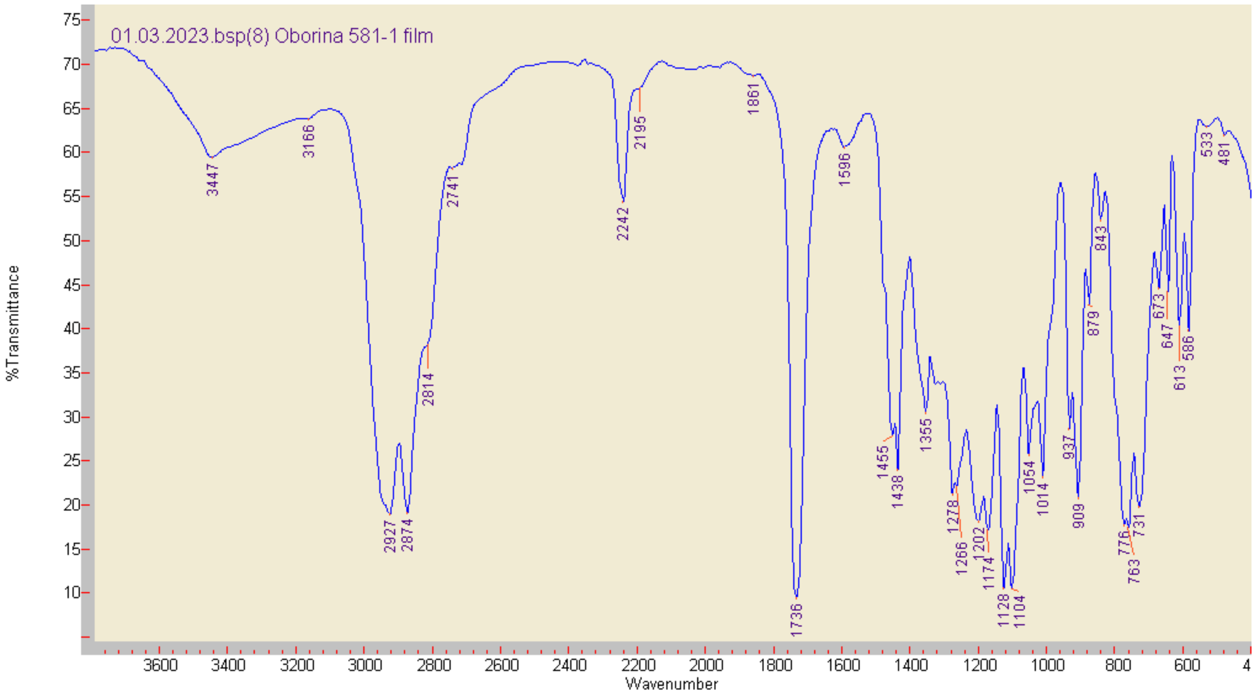

Fig. S4. IR spectrum of silatrane **10**

NMR spectra

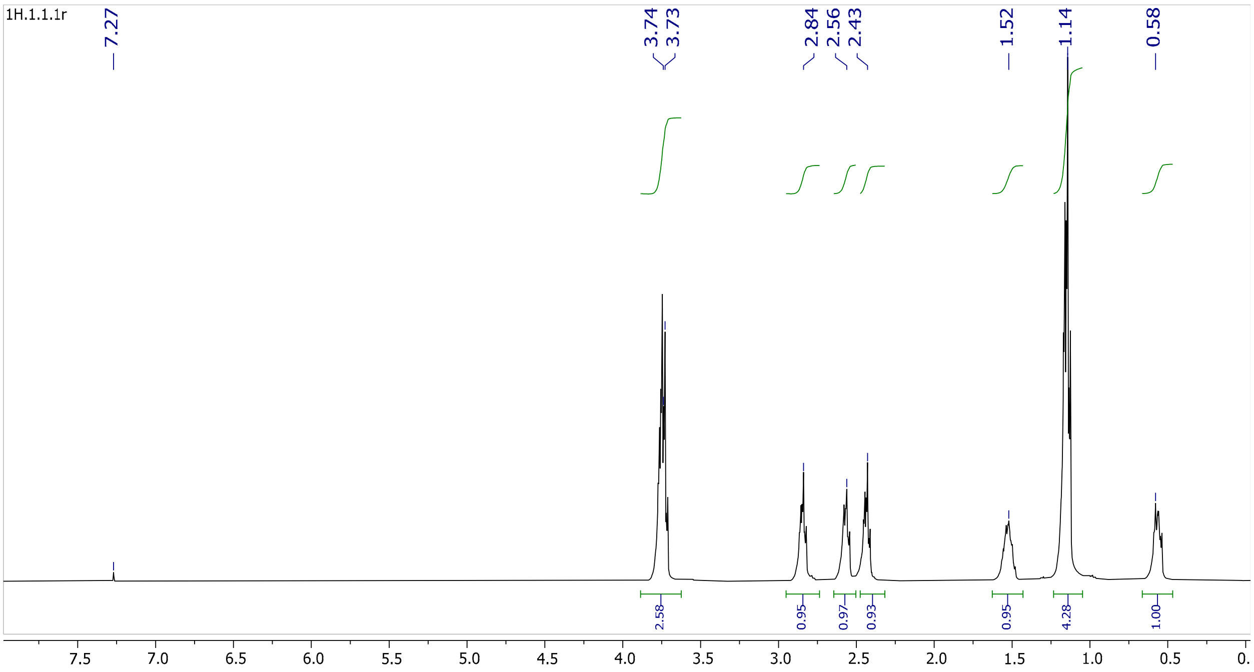

Fig. S5. ^{1}H NMR spectrum of silane **3**

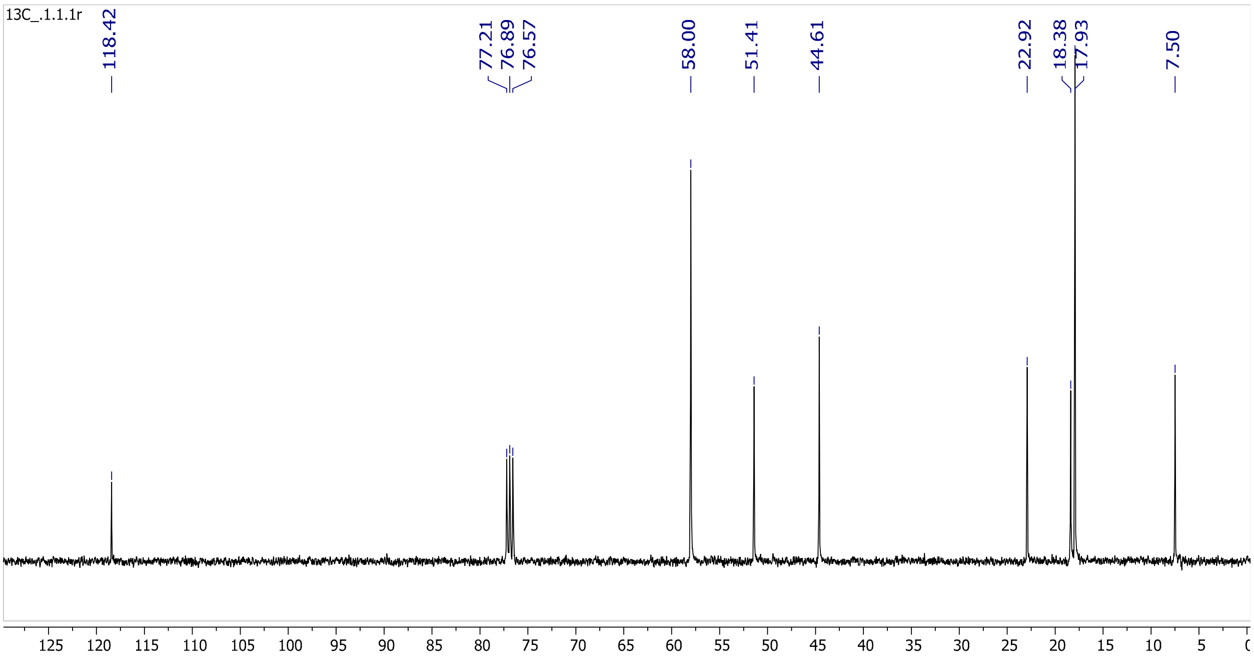

Fig. S6. ^{13}C NMR spectrum of silane **3**

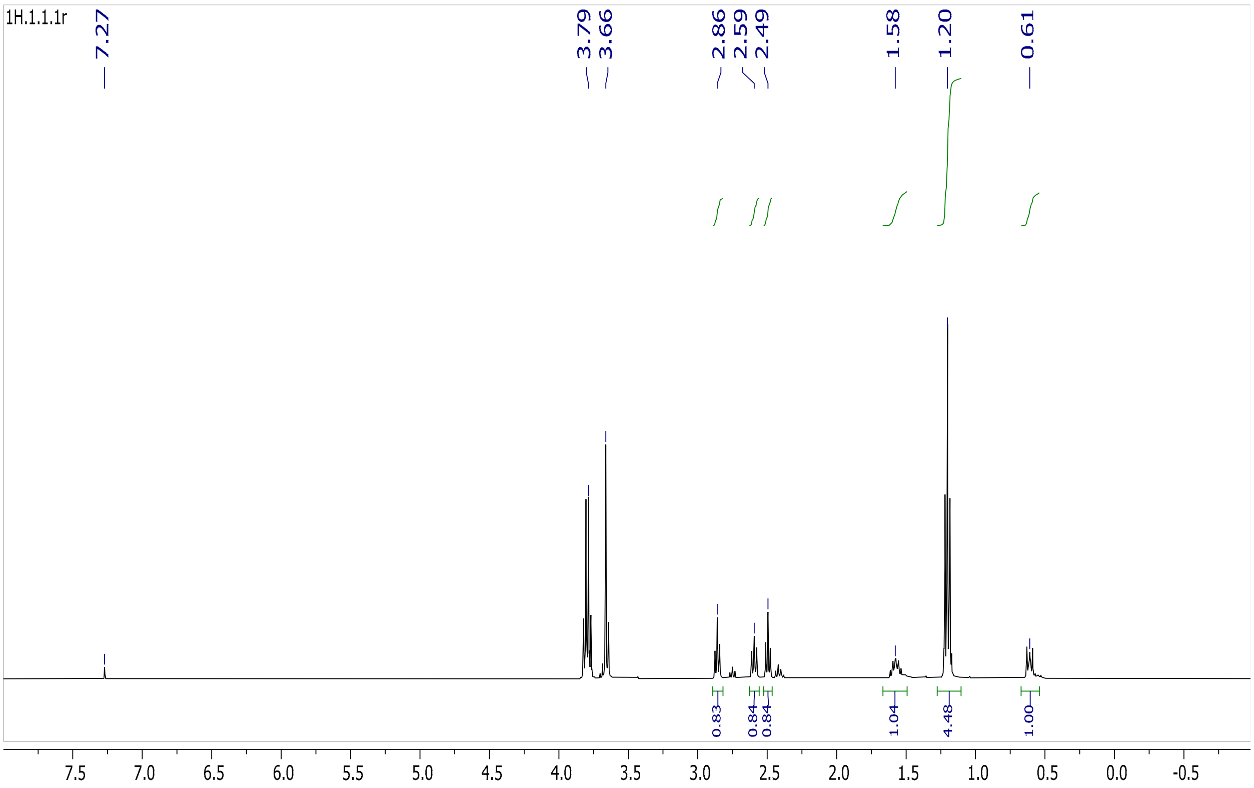

Fig. S7. ^{1}H NMR spectrum of silane **4**

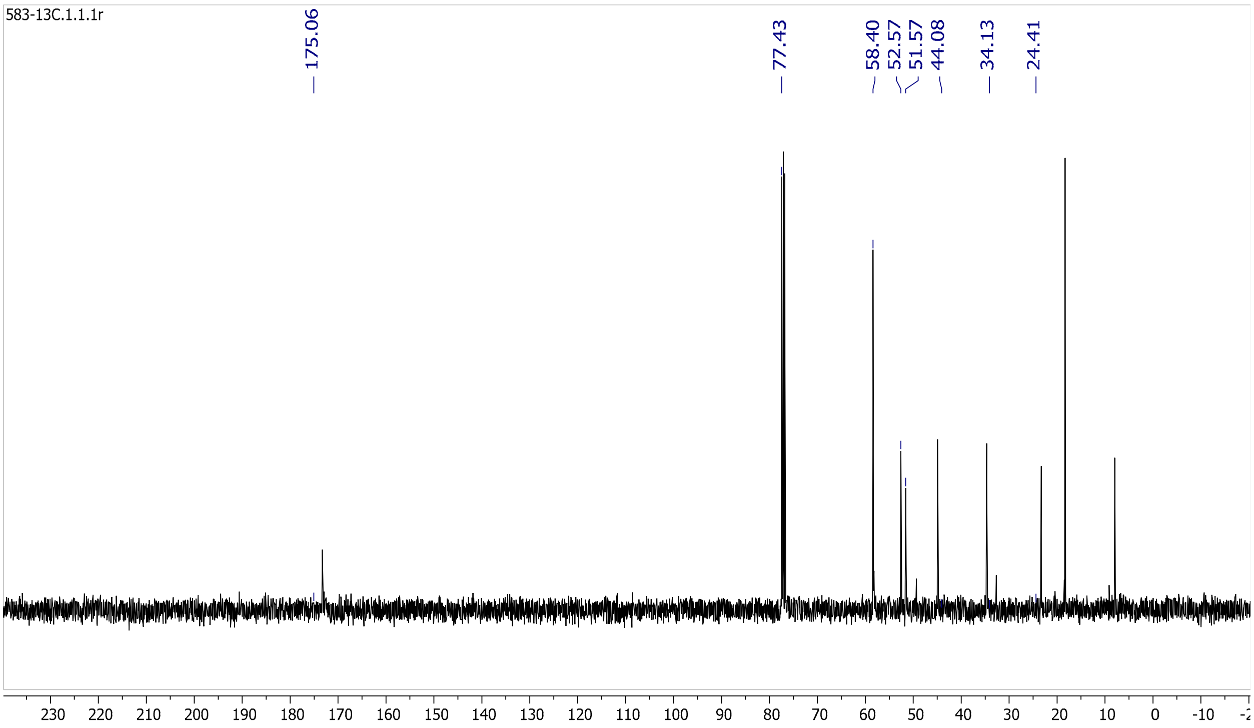

Fig. S8. ^{13}C NMR spectrum of silane **4**

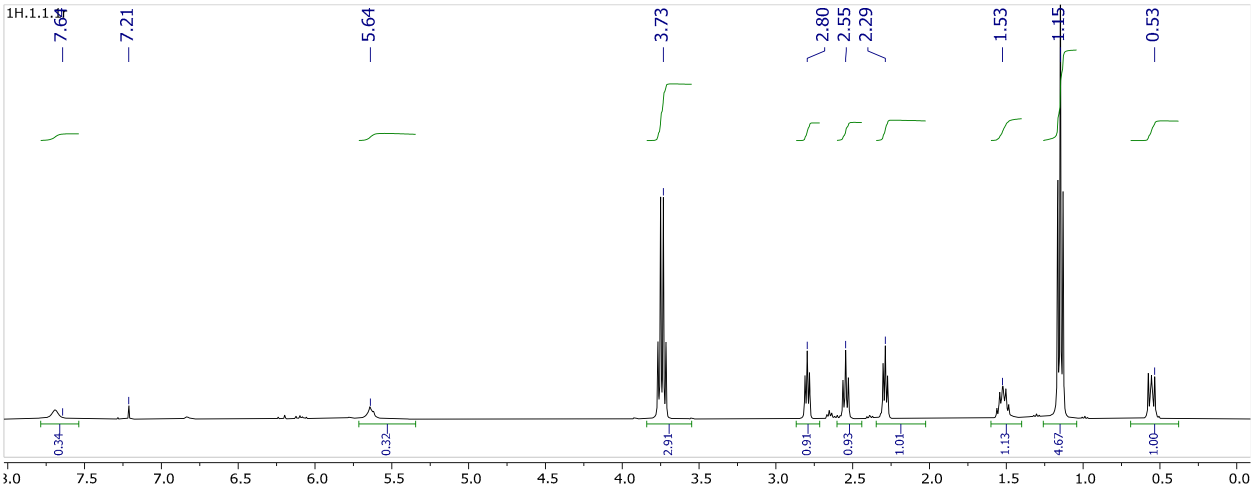

Fig. S9. ^{1}H NMR spectrum of silane **5**

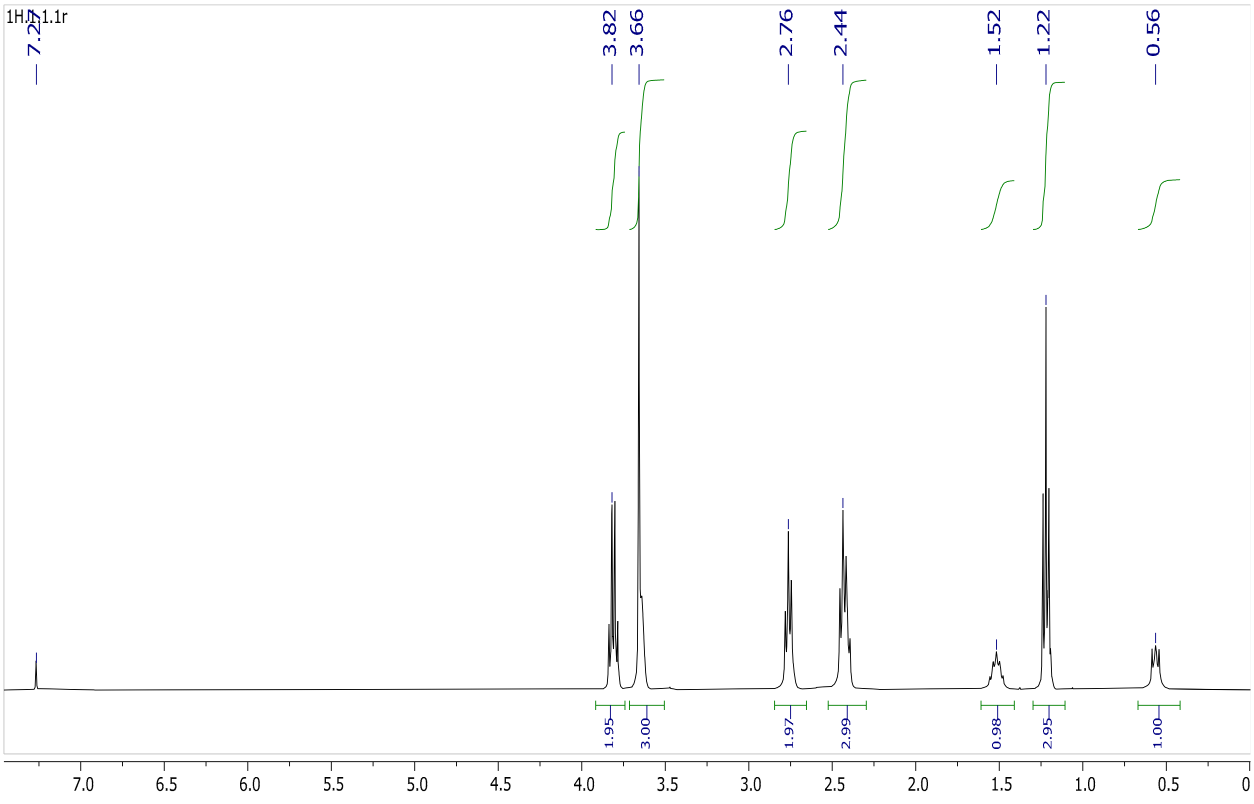

Fig. S10. ^{1}H NMR spectrum of silane **7**

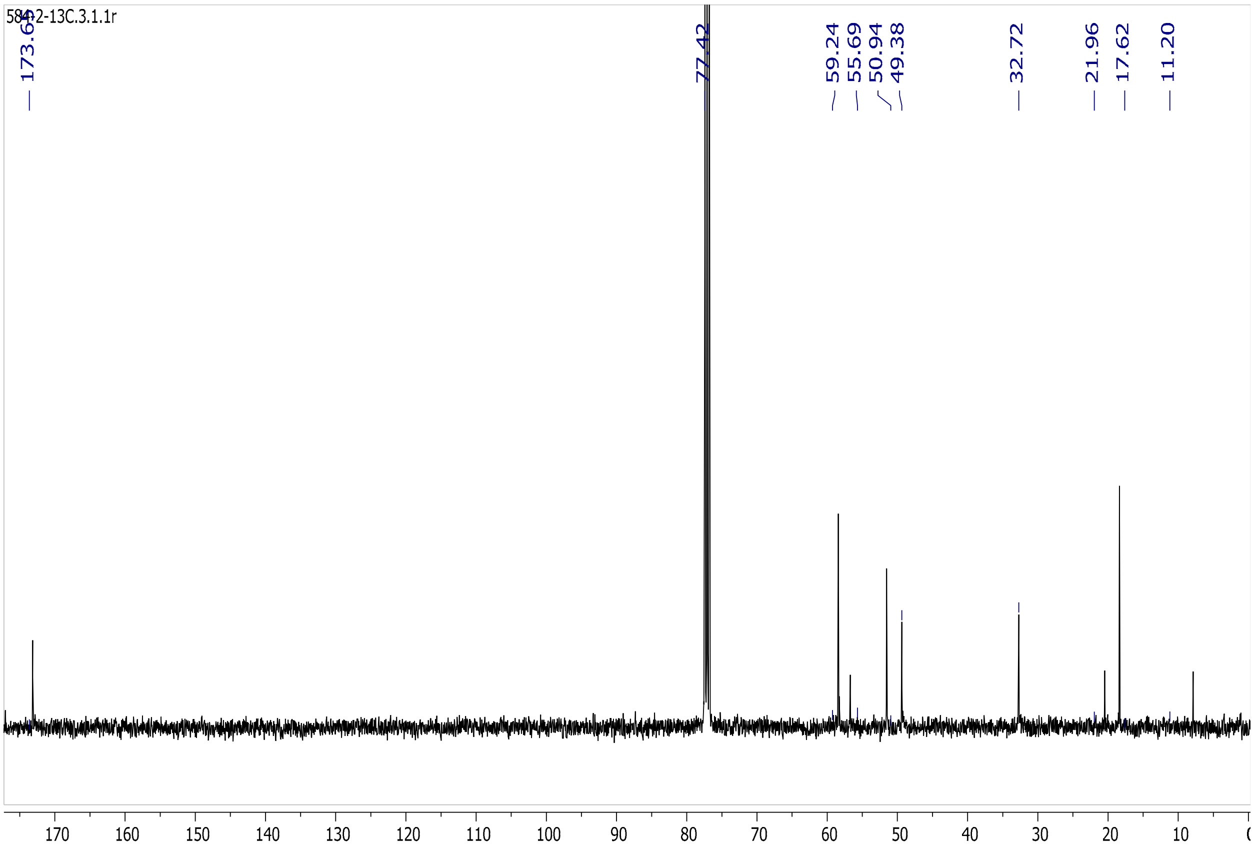

Fig. S11. ^{13}C NMR spectrum of silane **7**

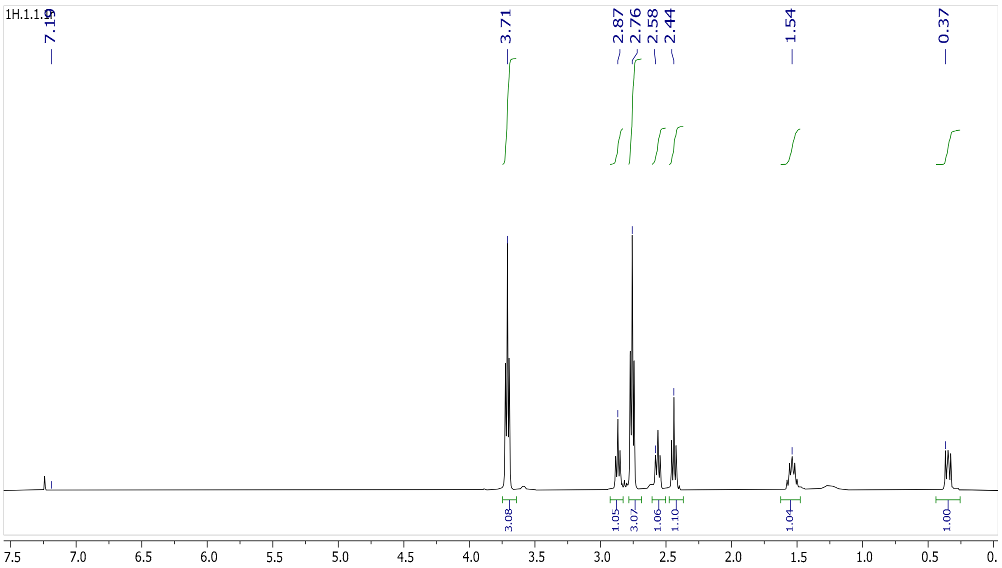

Fig. S12 ^{1}H NMR spectrum of silatrane **9**

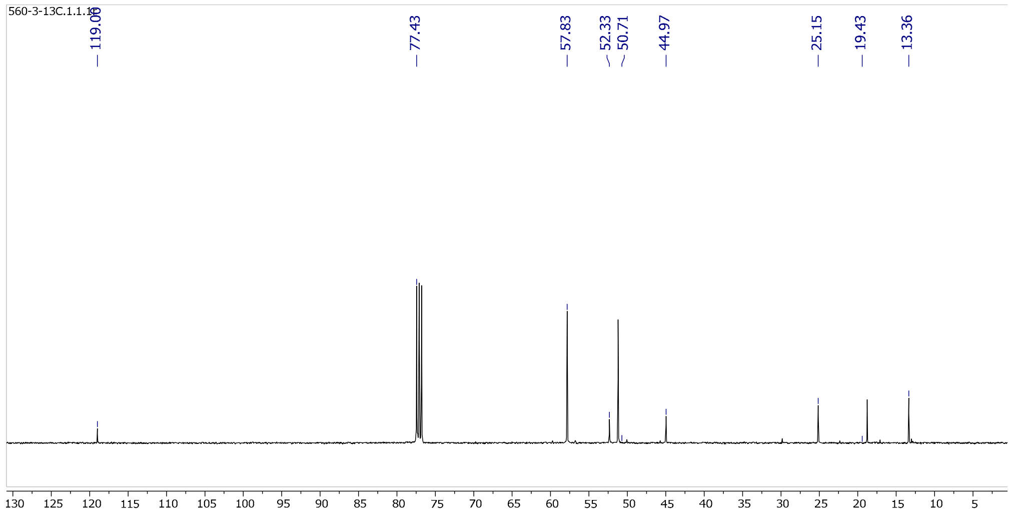

Fig. S13 ^{13}C NMR spectrum of silatrane **9**

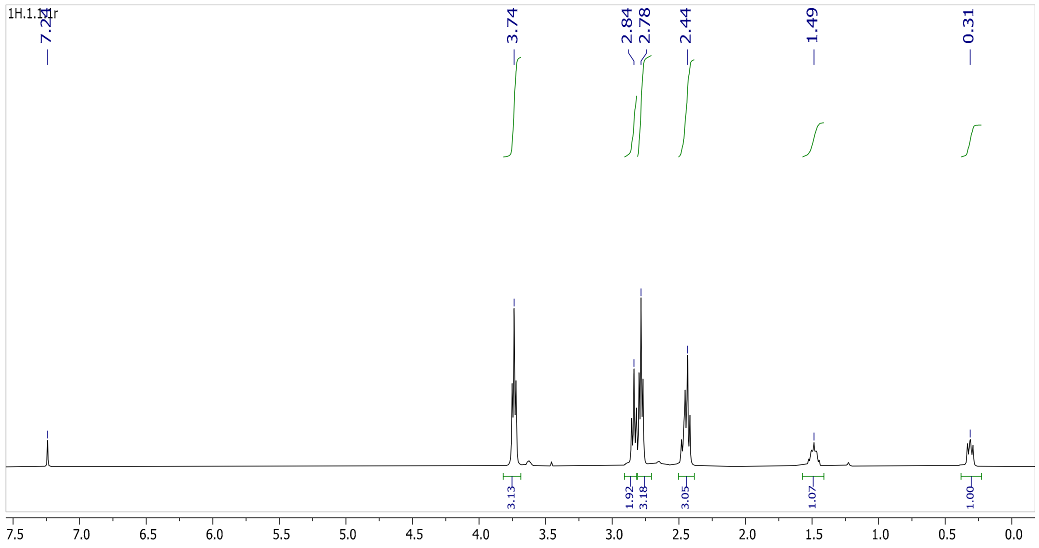

Fig. S14 ^{1}H NMR spectrum of silatrane **12**

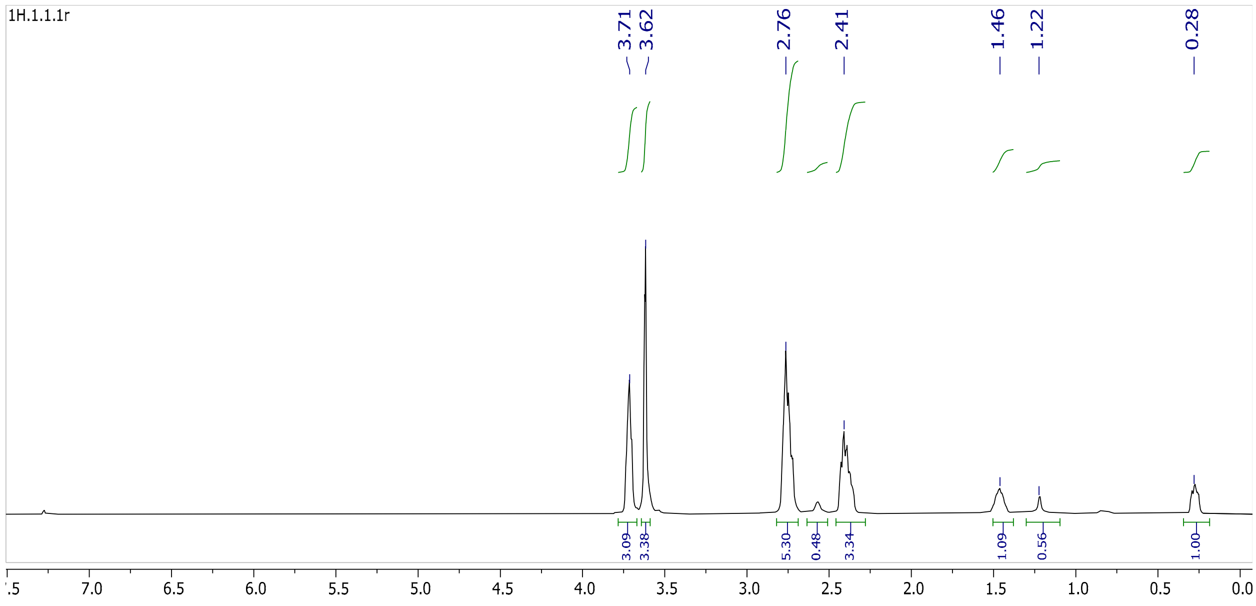

Fig. S15. ^{1}H NMR spectrum of silatrane **13**

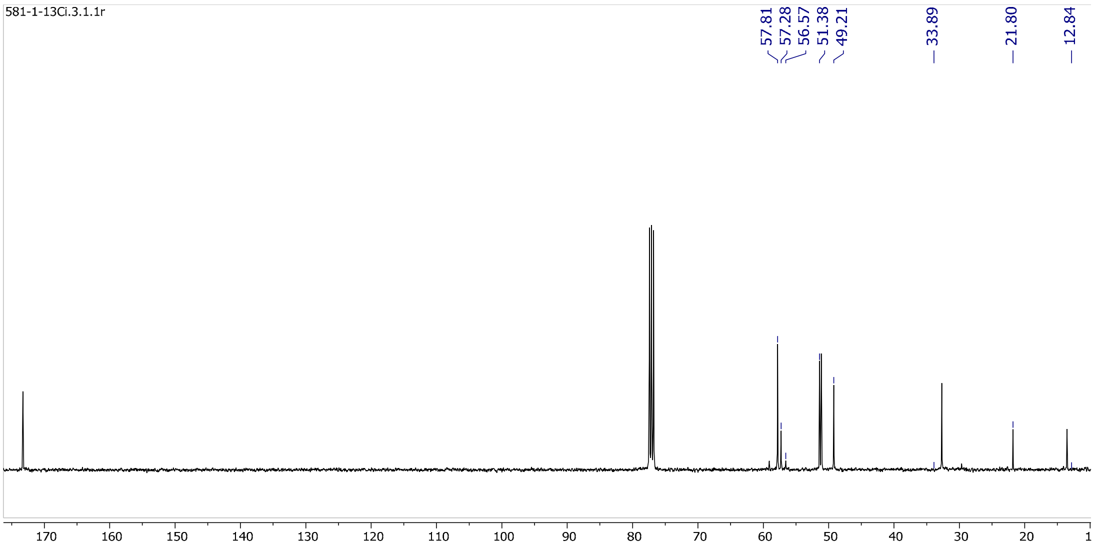

Fig. S16. ^{13}C NMR spectrum of silatrane **13**

Mass spectra

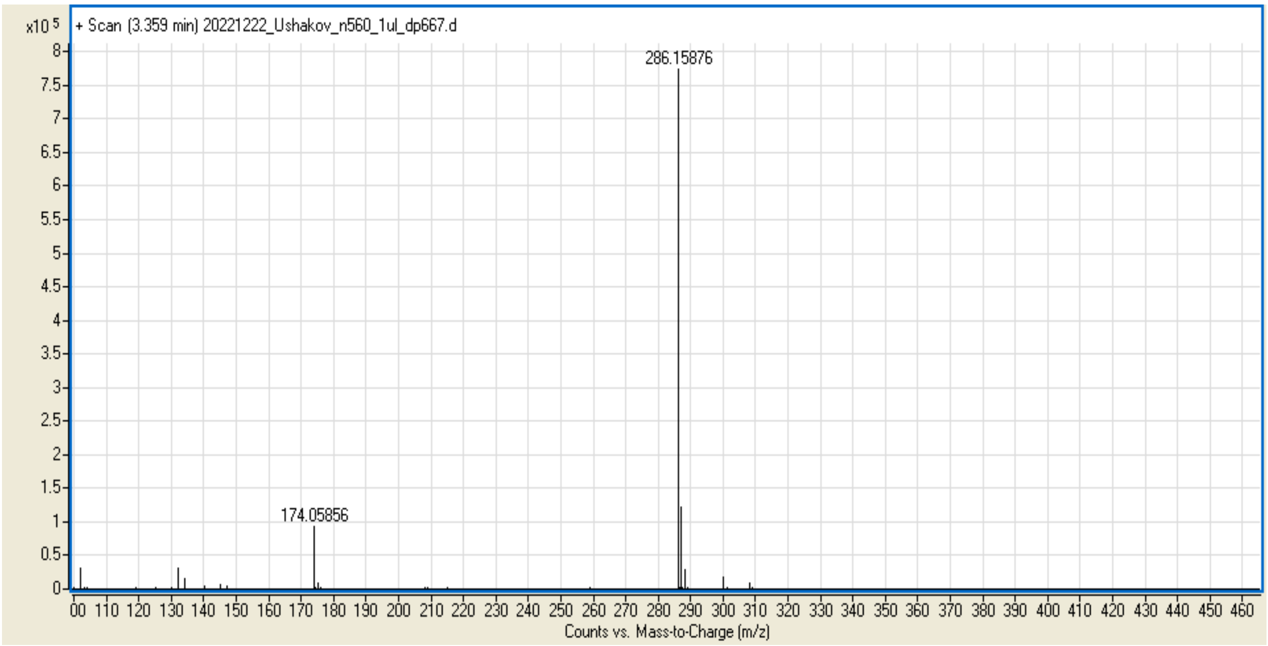

Fig. S17. Mass spectrum of silatrane **9**

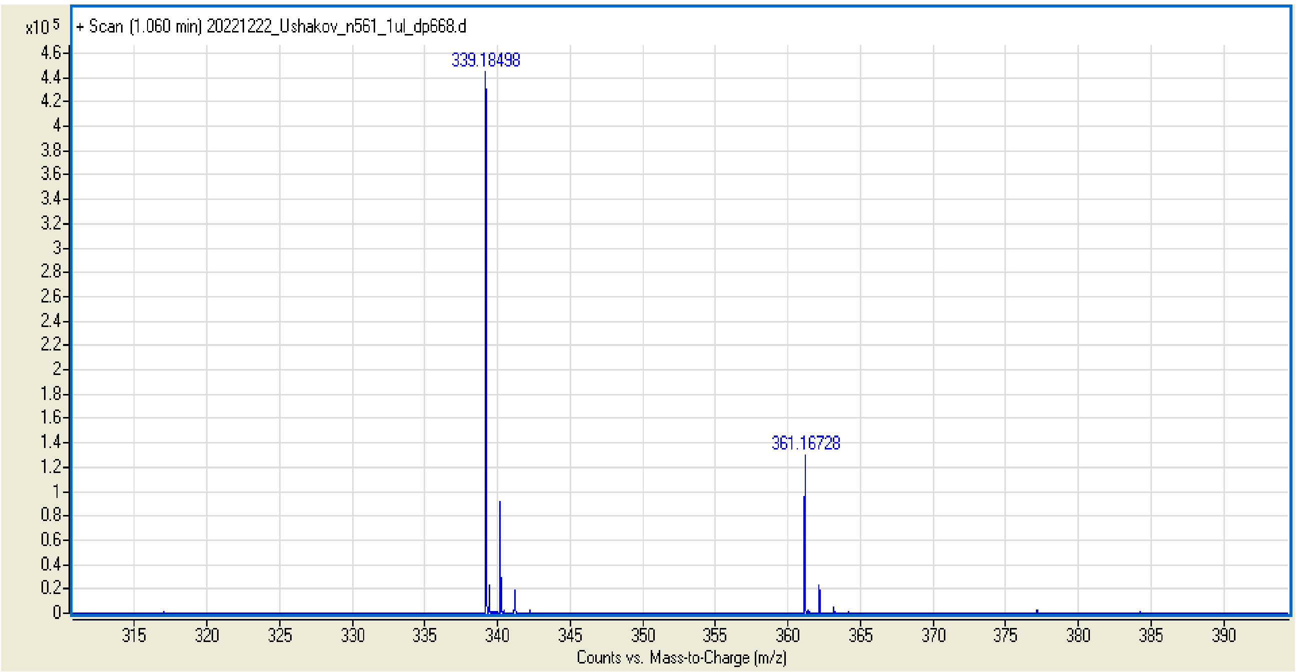

Fig. S18. Mass spectrum of siltrane **12**

X-ray crystallography

Table S1. Crystal data and structure refinement for compounds **9** and **12**

Work identification code	irich_560_s1_150K	irich_565_rem1_s1_150K
Compound	**9**	**12**
Empirical formula	$C_{12}H_{23}N_3O_3Si$	$C_{15}H_{26}N_4O_3Si$
Formula weight, g/mol	285.42	338.49
Temperature, K	150(2)	150(2)
Radiation	MoKα (λ = 0.71073)	MoKα (λ = 0.71073)
Crystal system	monoclinic	orthorhombic
Space group	$P2_1/c$	*Pbca*
a, Å / α, °	11.1251(3) / 90	12.2082(3) / 90
b, Å / β, °	10.2541(2)/ 110.5460(10)	13.5352(4) / 90
c, Å / γ, °	13.4672(3) / 90	21.5488(6) / 90
Volume, Å^3	1438.59(6)	3560.73(17)
Z	4	8
ρ_{calc}, g/cm^3	1.318	1.263
μ, mm^{-1}	0.172	0.152
F(000)	616.0	1456.0
Crystal size, mm^3	0.17 × 0.12 × 0.025	0.34 × 0.33 × 0.29
2θ range, °	3.9 – 63.1	4.9 – 63.0
Index ranges	−14 ≤ h ≤ 16, −11 ≤ k ≤ 15, −19 ≤ l ≤ 18	−17 ≤ h ≤ 17, −18 ≤ k ≤ 19, −31 ≤ l ≤ 31
Reflections collected / independent	14251 / 4769	89579 / 5916
R_{int} / R_σ	0.0342 / 0.0411	0.0351 / 0.0139

Data / restraints / parameters	4769 / 0 / 175	5916 / 45 / 237
Goodness-of-fit on F^2	1.036	1.036
Final R_1 / wR_2 indexes I>=2σ (I)<br>all data	0.0404 / 0.0945<br>0.0564 / 0.1024	0.0309 / 0.0859<br>0.0354 / 0.0890
Largest diff. peak / hole, e/Å^3	0.41 / −0.32	0.35 / −0.26

X-ray crystallography. Single crystals of **9** and **12** were grown. The molecular structures are depicted in Fig. S19. The selected bonds lengths and angles are given in Table S2.

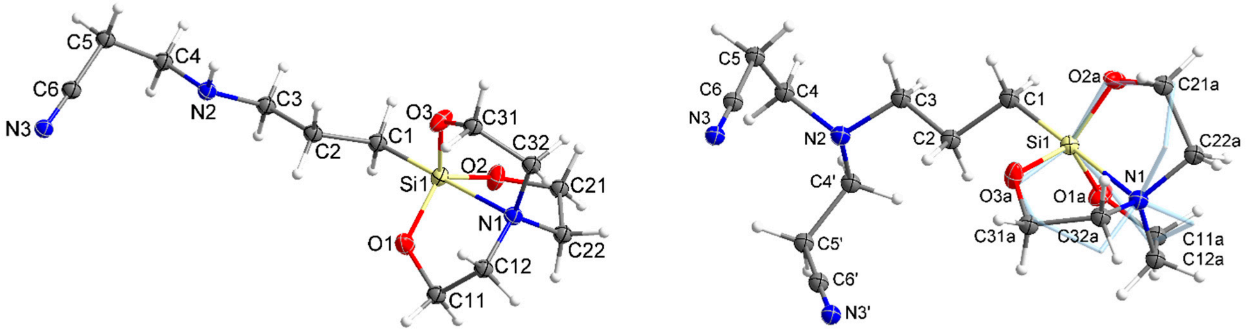

Fig. S19. Molecular structure of compounds **9** (left) and **12** (right). Atomic displacements for non-hydrogen atoms are shown at 50% probability. Alternative conformation of silatrane fragment (Oxb, Cxxb) of **12** is shown with pale blue sticks.

Table S2. Selected bond lengths and angles for compounds **2a** and **2b**. Estimated standard deviations for the bonds and angles not exceed 2·10^{-3}Å and 0.15°.

Bond lengths, Å	**9**	**12**
Si1–N1	2.203	2.181
Si1–C1	1.875	1.888
Si1–O*x*	1.663-1.669	1.677-1.678
Bond angles, °		
C1–Si1–O*x*	97.1-98.0	97.0-97.7
O*x*–Si–O*x*′	117.9-118.5	117.3-119.5
N1–Si1–O*x*	81.9-82.6	82.3-83.0
Si1–O*x*–C*x*1	122.6-123.4	121.8-123.6
O*x*–C*x*1–C*x*2	108.3-109.3	107.9-109.3
N1–C*x*2–C*x*1	105.9-106.1	105.4-106.3

* Geometrical parameters of the major conformation only.

Initial data from Olex2.

Bond lengths, Å	**9**	**12**
Si1–N1	2.2034(10)	2.1813(7)
Si1–C1	1.8748(12)	1.8878(8)
Si1–O*x*	1.6684(9)<br>1.6634(9)<br>1.6686(9)	1.6784(17)<br>1.6775(17)<br>1.6769(13)

Bond angles, °		
C1–Si1–O*x*	98.35(5)<br>97.07(5)<br>97.97(5)	97.04(6)<br>97.65(6)<br>97.20(5)
O*x*–Si–O*x*′	118.50(5)<br>117.87(5)<br>118.21(5)	118.49(8)<br>119.46(7)<br>117.29(7)
N1–Si1–O*x*	82.09(4)<br>82.63(4)<br>81.89(4)	82.75(6)<br>83.02(5)<br>82.35(5)
Si1–O*x*–C*x*1	123.17(8)<br>122.57(8)<br>123.43(8)	122.95(13)<br>121.82(13)<br>123.58(10)
O*x*–C*x*1–C*x*2	108.25(10)<br>109.30(10)<br>108.97(10)	107.87(13)<br>109.33(14)<br>108.83(14)
N1–C*x*2–C*x*1	106.00(10)<br>106.10(9)<br>105.88(10)	106.13(8)<br>105.35(8)<br>106.25(9)

Spectrophotometric determination of the elements

A 50-mg sample of the polymer was intensely mixed with 50 mL of a solution of a metal ion in hydrochloric (for Ni, Cu) or nitric (for silver) acid. The acid concentration varied between 0.5 and 0.6 mol/L, and the metal ion content ranged from 0.05 to 0.8 mg/mL. At the end of the experiment, the polymer was separated from the solution by filtration and washed on the filter with distilled water. The wash water was combined with the main filtrate, and the residual metal content was determined in them by spectrophotometric method.

Modification of hydroxylated surfaces

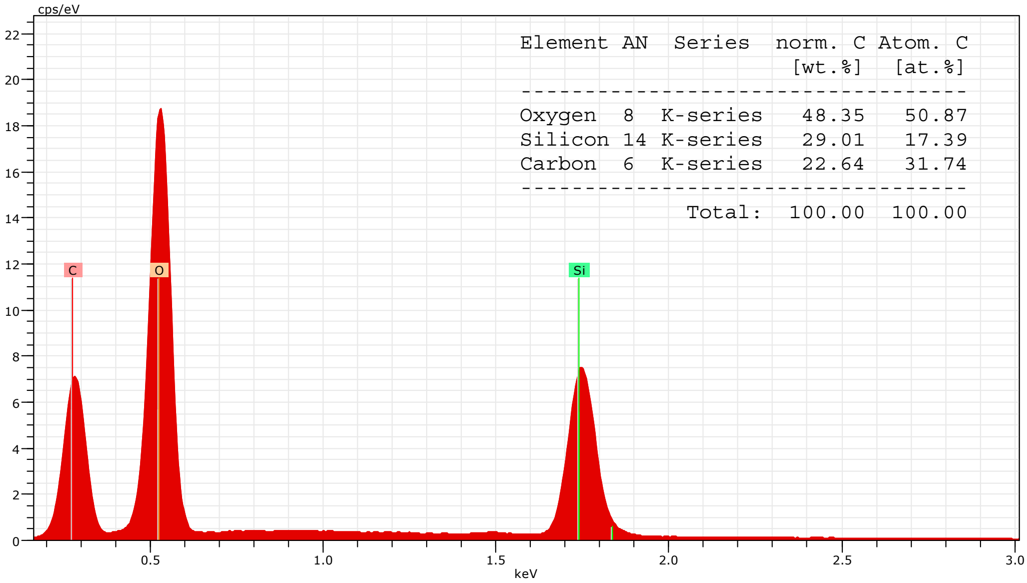

Fig. S20. EDX spectrum and elemental composition of modified silica gel (**S7**)

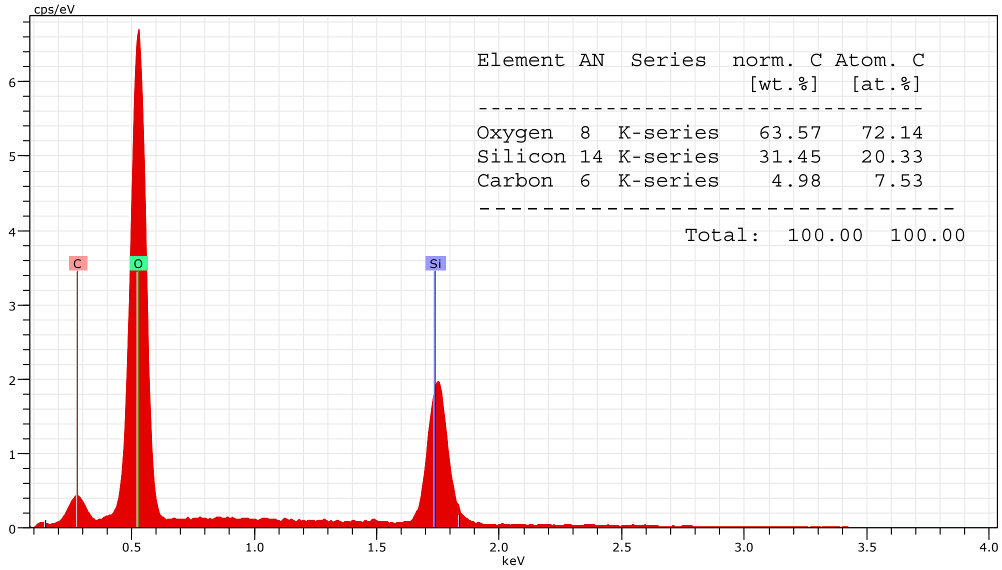

Fig. S21. EDX spectrum and elemental composition of initial glass (**G**)

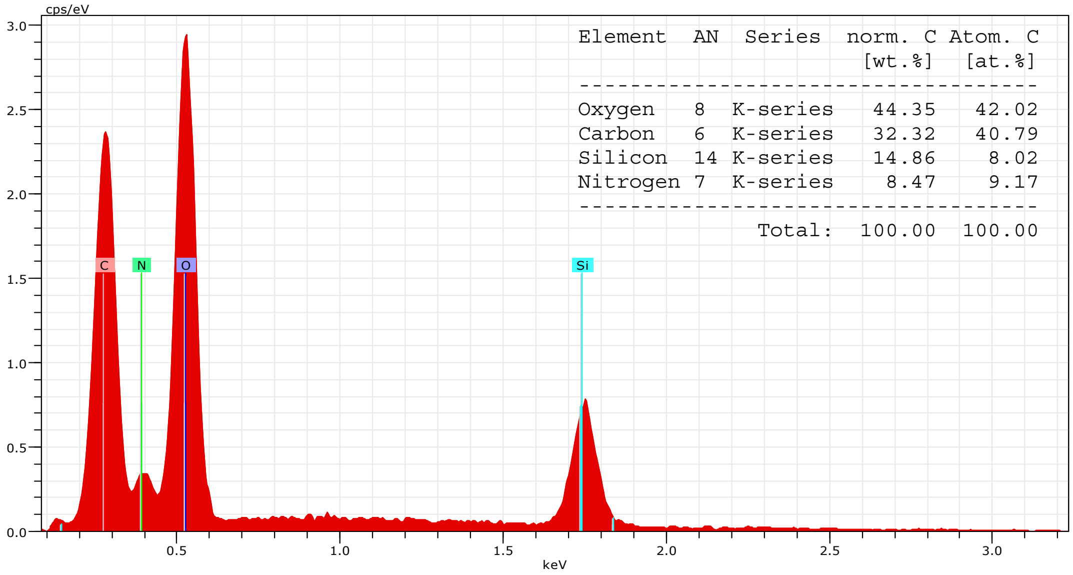

Fig. S22. EDX spectrum and elemental composition of modified glass (**G4**)

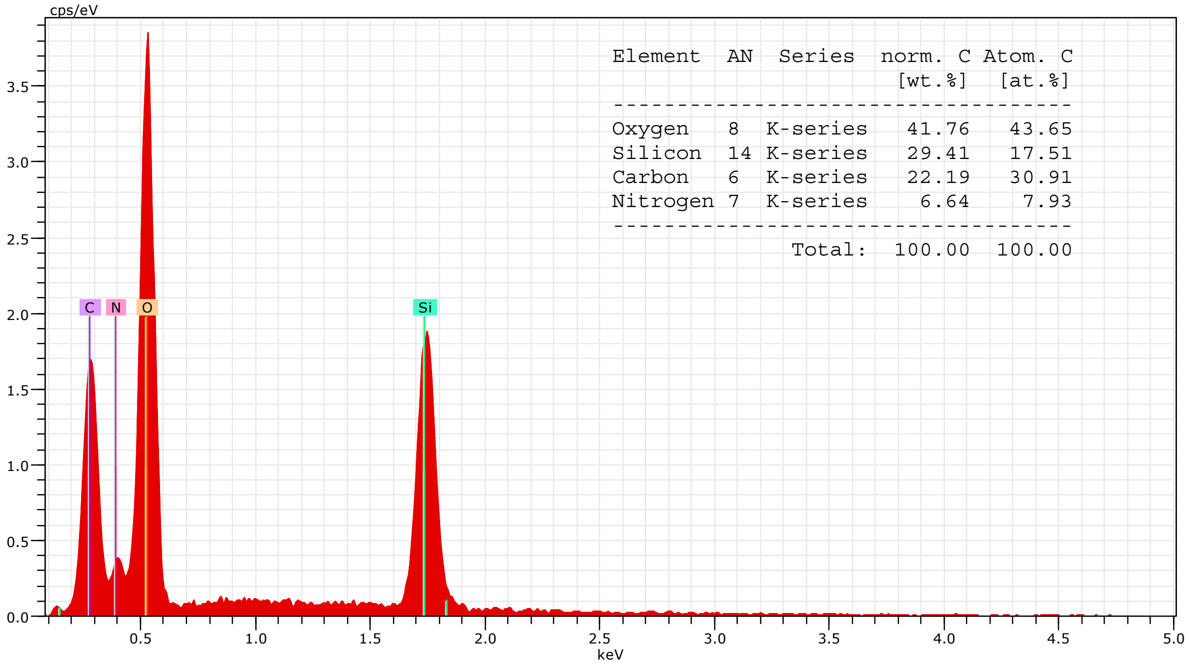

Fig. S23. EDX spectrum and elemental composition of modified glass (**G5**)

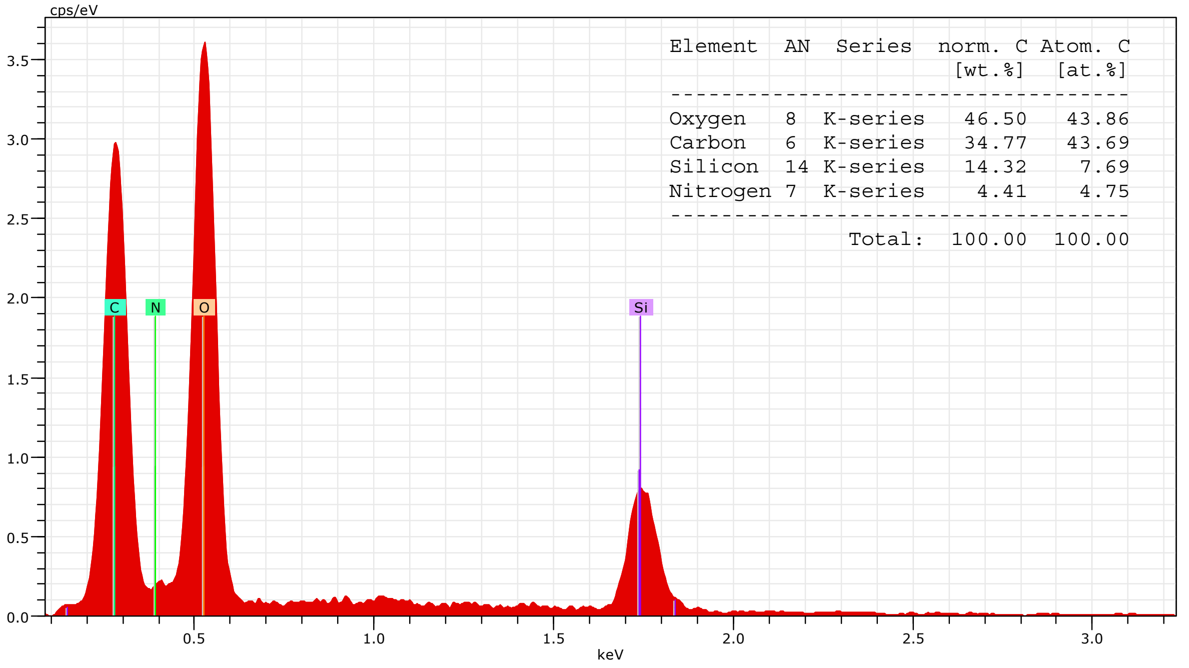

Fig. S24. EDX spectrum and elemental composition of modified glass (**G7**)

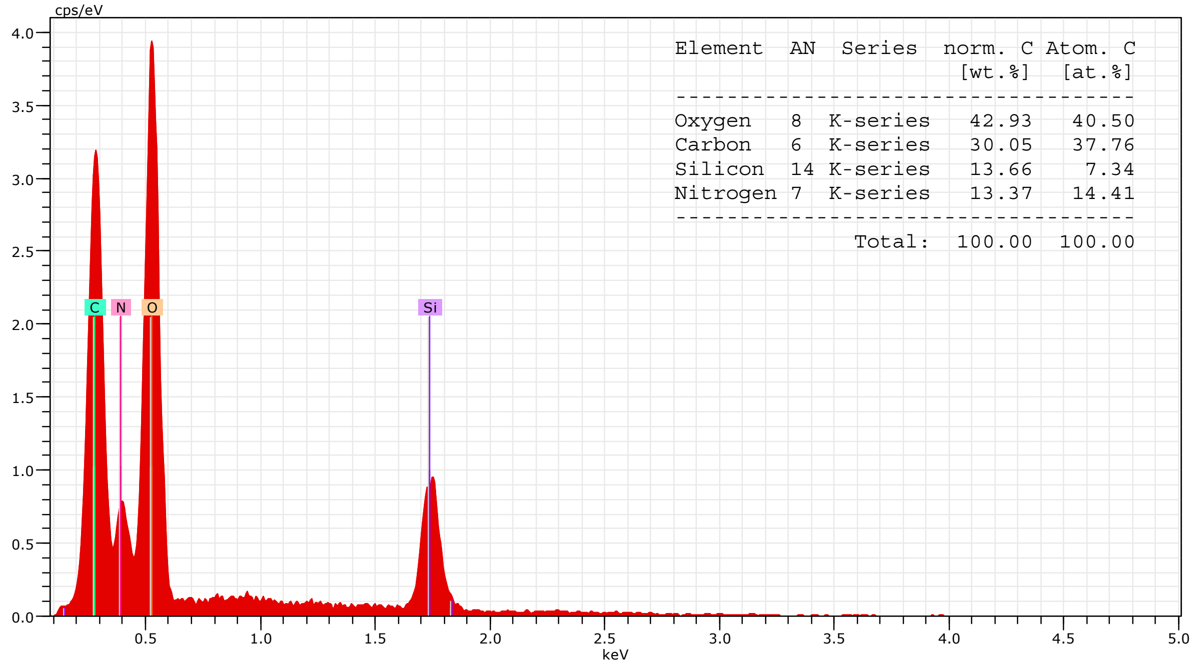

Fig. S25. EDX spectrum and elemental composition of modified glass (**G8**)

Adsorption

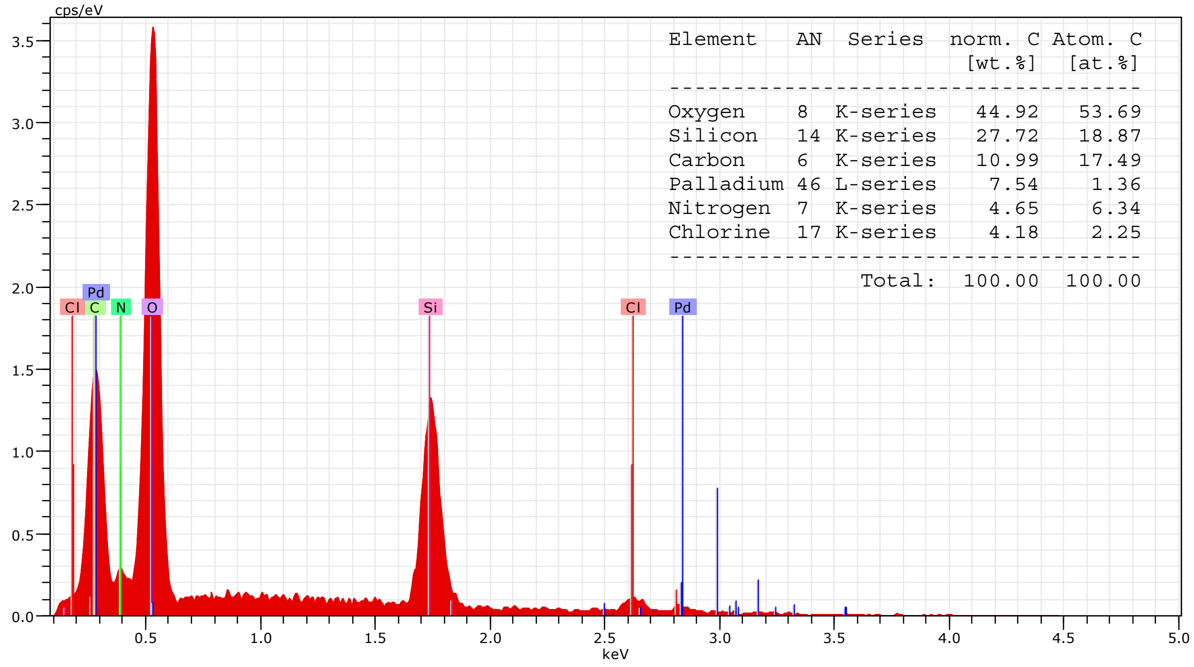

Fig. S26. EDX spectrum and elemental composition of (**G4+Pd**)

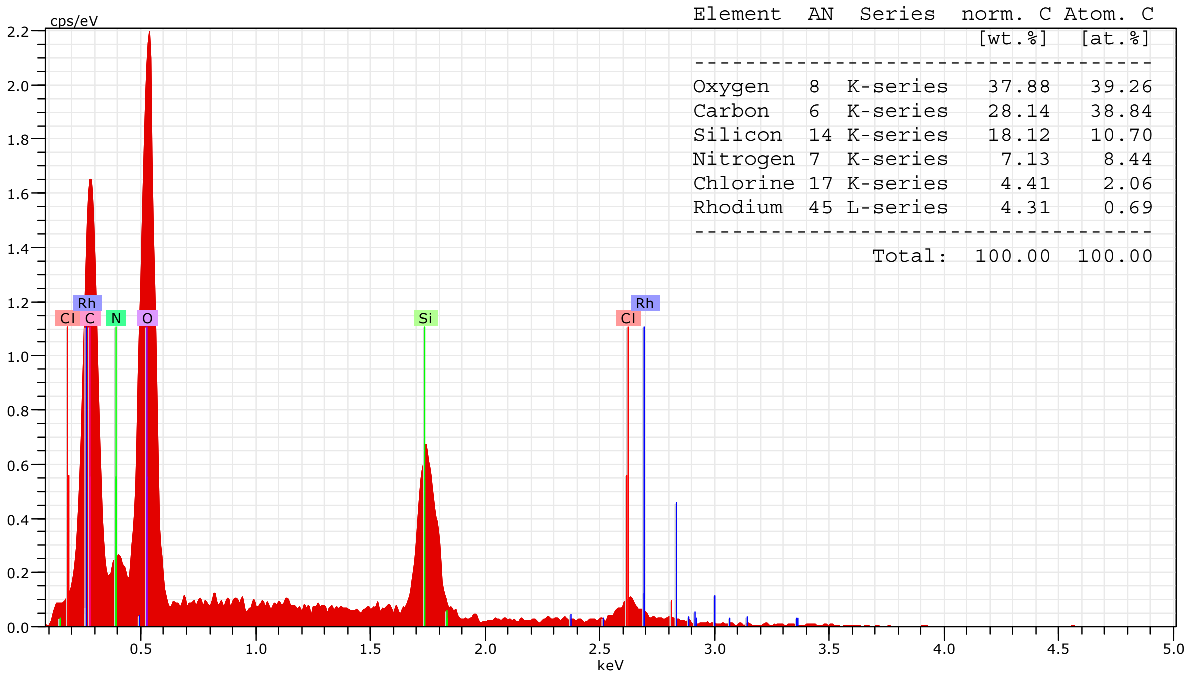

Fig. S27. EDX spectrum and elemental composition of (**G4+Rh**)

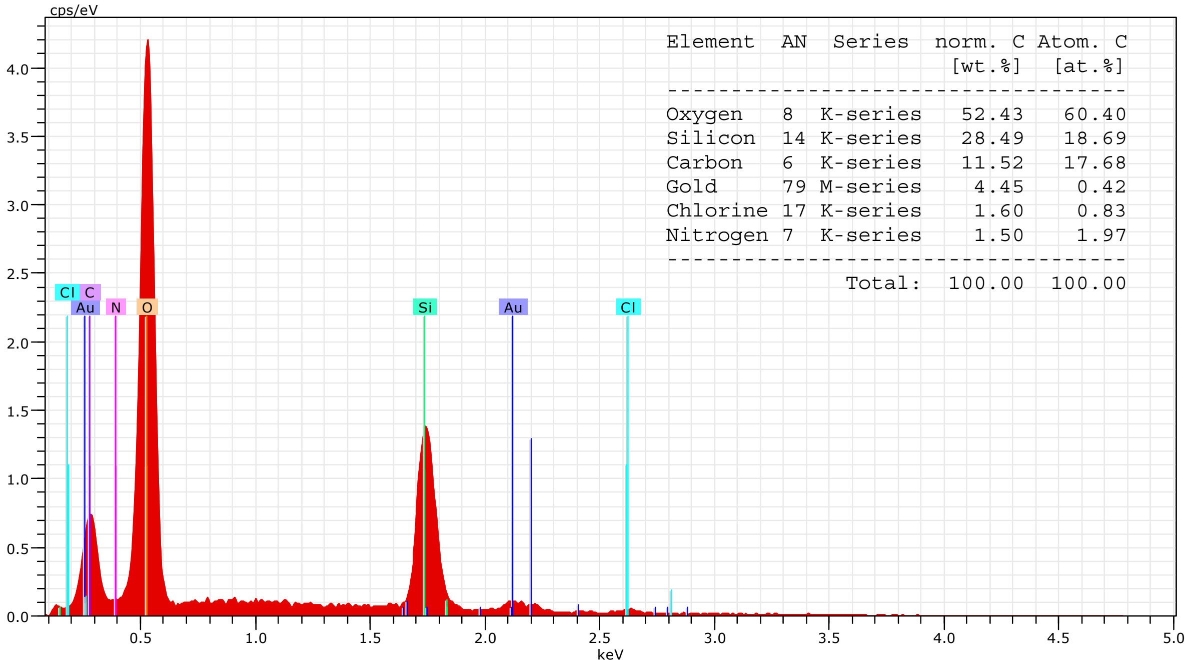

Fig. S28. EDX spectrum and elemental composition of (**G7+Au**)

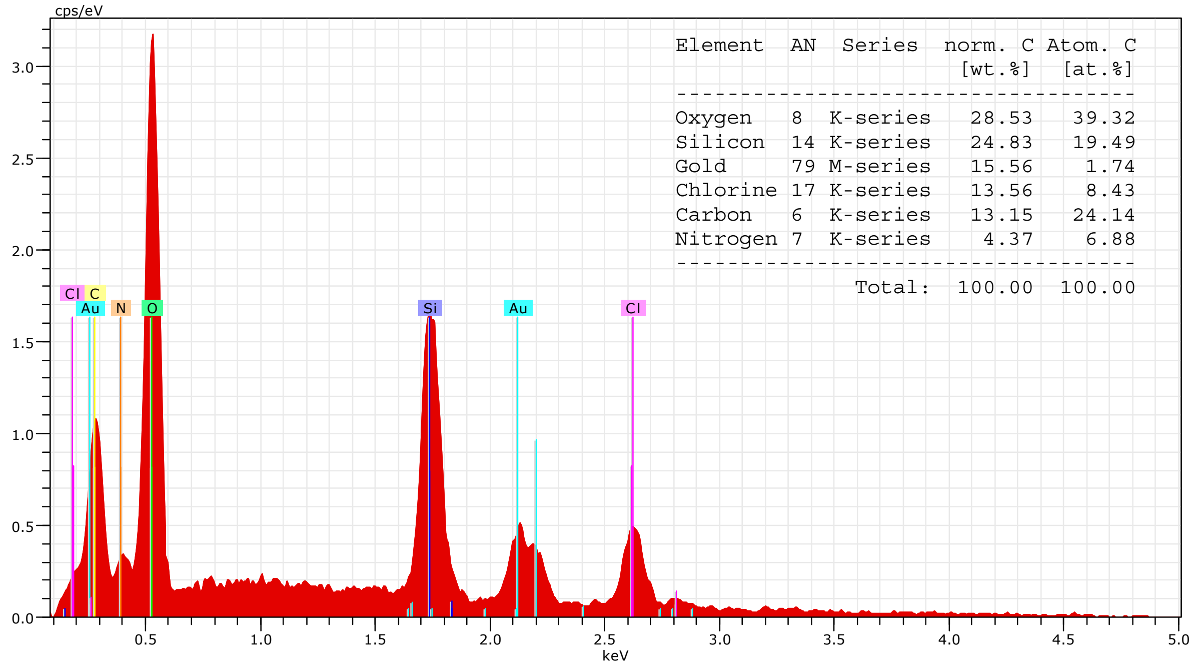

Fig. S29. EDX spectrum and elemental composition of (**G5+Au**)

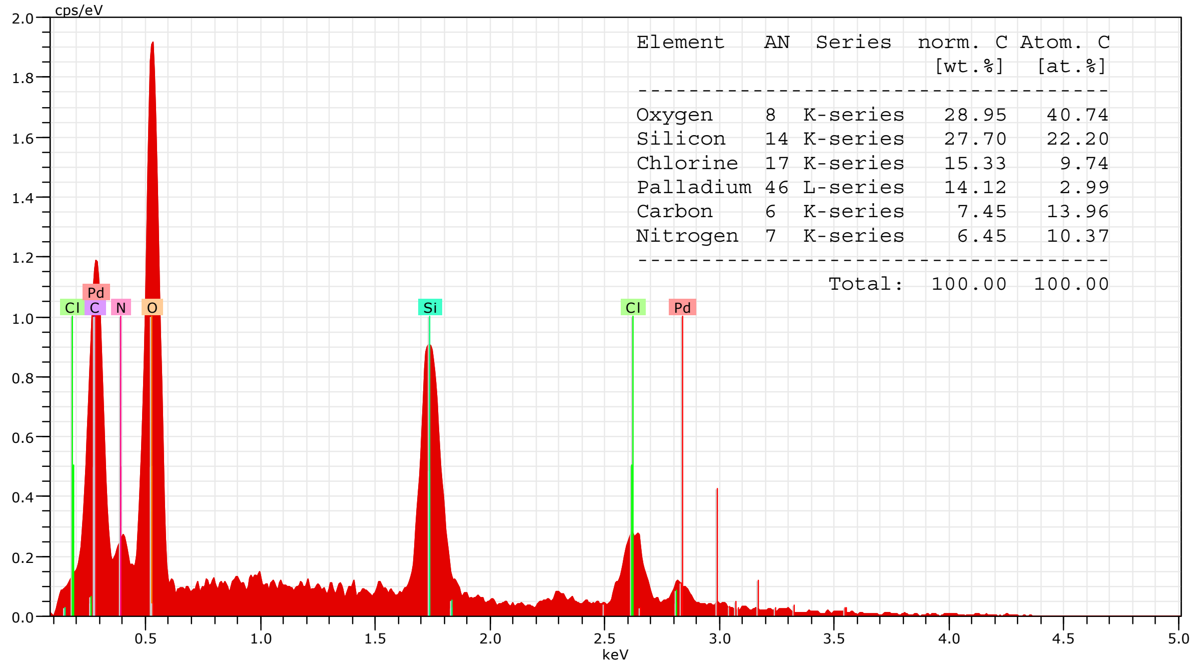

Fig. S30. EDX spectrum and elemental composition of (**G5+Pd**)

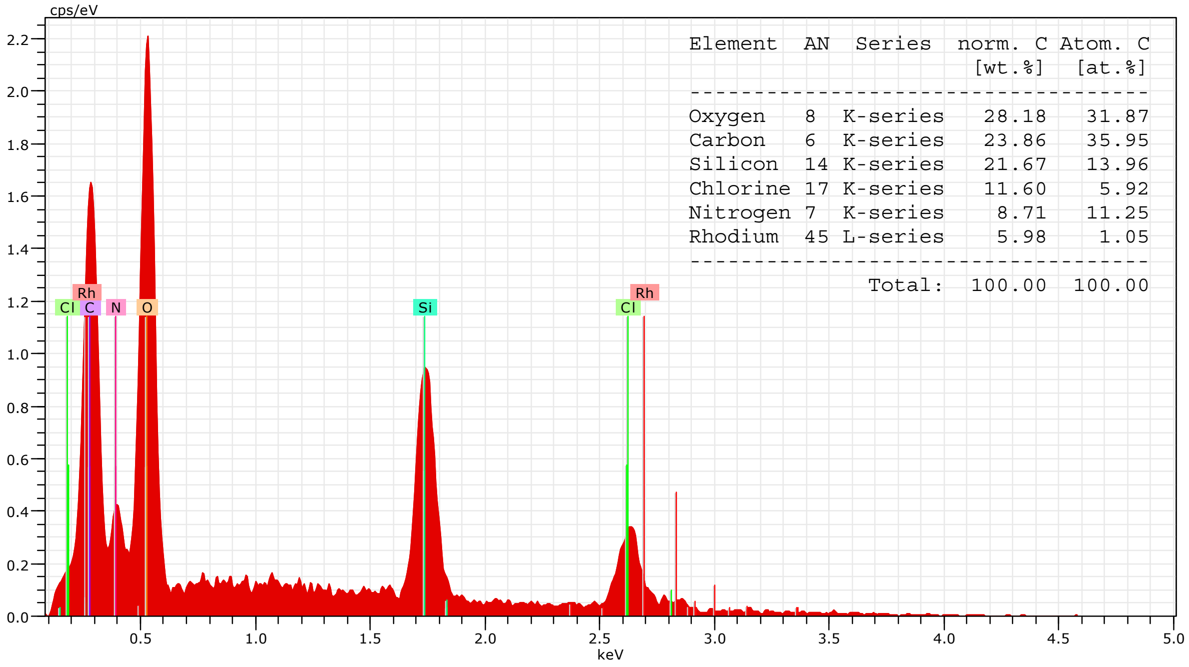

Fig. S31. EDX spectrum and elemental composition of (**G5+Rh**

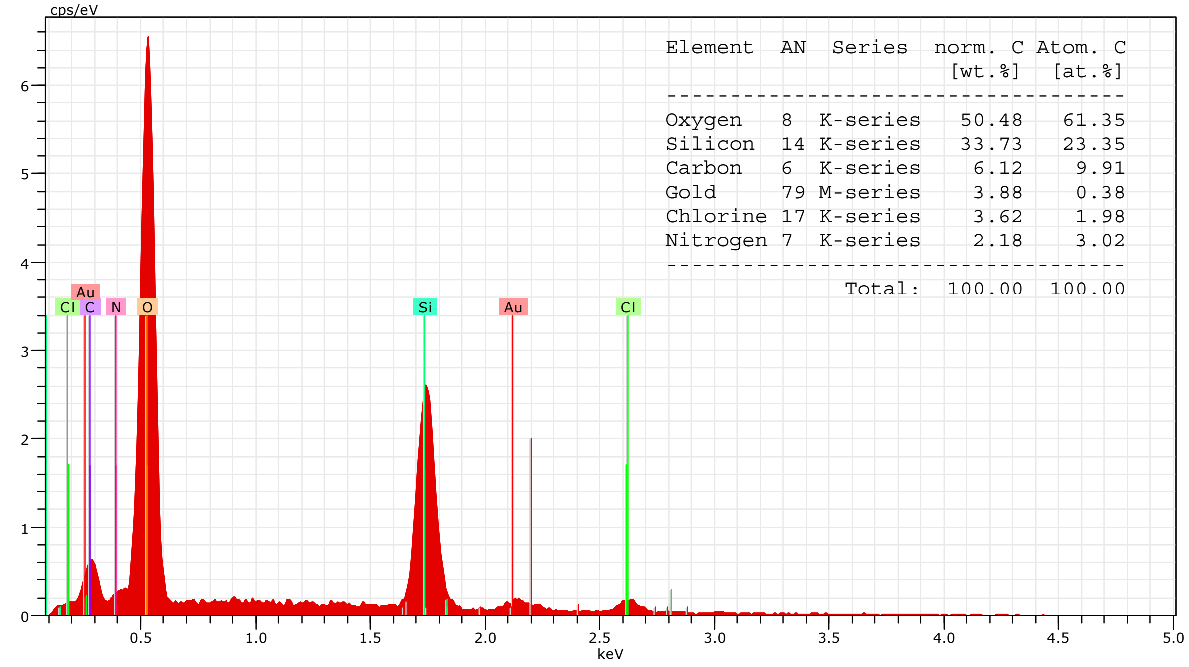

Fig. S32. EDX spectrum and elemental composition of (**G8+Au**)

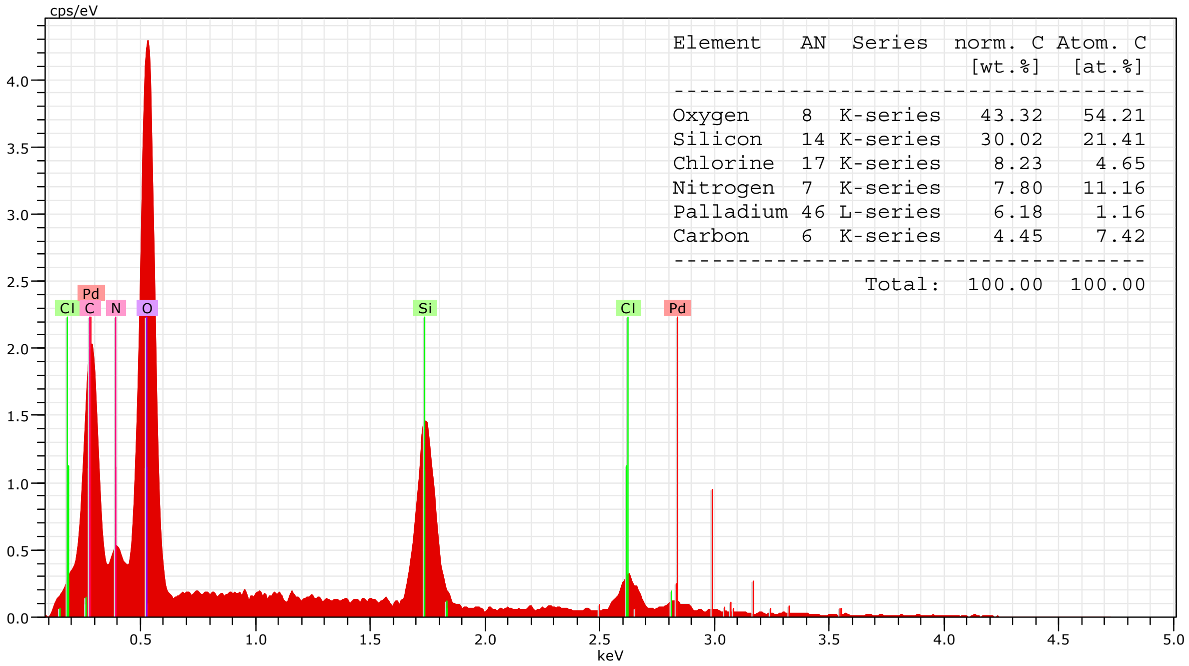

Fig. S33. EDX spectrum and elemental composition of (**G8+Pd**)

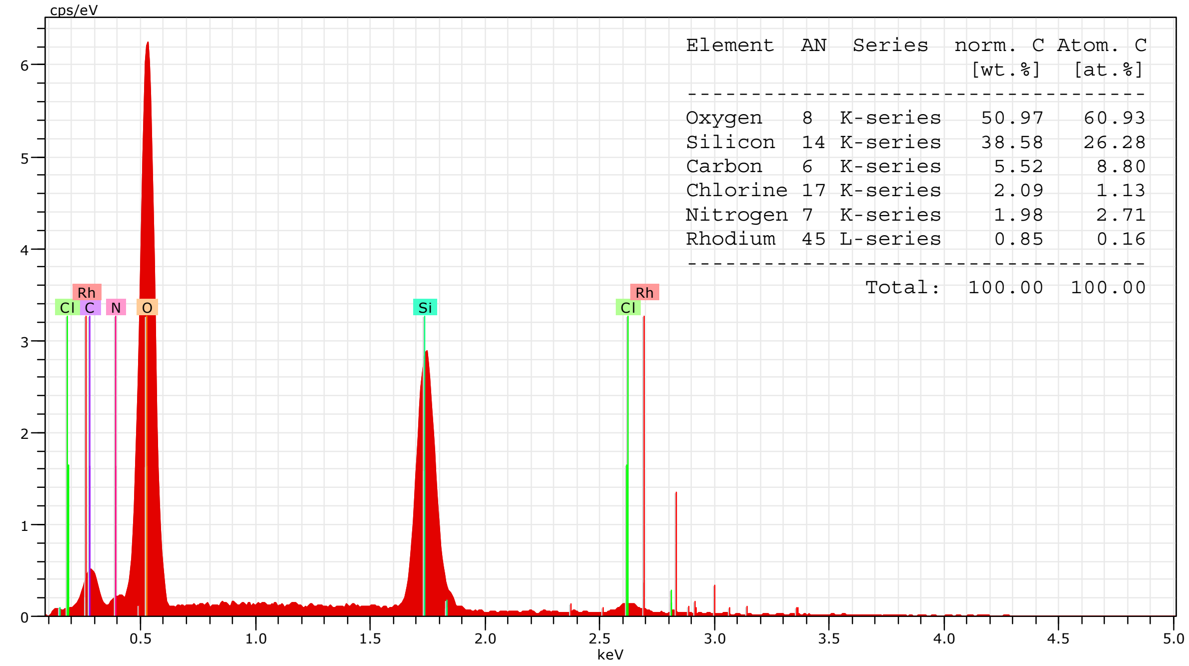

Fig. S34. EDX spectrum and elemental composition of (**G8+Rh**)
